# Lasting response by vertical inhibition with cetuximab and trametinib in *KRAS*‐mutated colorectal cancer patient‐derived xenografts

**DOI:** 10.1002/1878-0261.13510

**Published:** 2023-09-03

**Authors:** Timm M. Reissig, Swetlana Ladigan‐Badura, Anja Steinberg, Abdelouahid Maghnouj, Ting Li, Berlinda Verdoodt, Sven T. Liffers, Michael Pohl, Heiner Wolters, Christian Teschendorf, Richard Viebahn, Jakob Admard, Nicolas Casadei, Andrea Tannapfel, Wolff Schmiegel, Stephan A. Hahn, Deepak B. Vangala

**Affiliations:** ^1^ Department of Molecular GI Oncology, Faculty of Medicine Ruhr‐University Bochum Germany; ^2^ Department of Medical Oncology, West German Cancer Center University Hospital Essen Germany; ^3^ Bridge Institute of Experimental Tumor Therapy, West German Cancer Center University Hospital Essen, University Duisburg‐Essen Germany; ^4^ Center for Hemato‐Oncological Diseases University Hospital Knappschaftskrankenhaus, Ruhr‐University Bochum Germany; ^5^ Institute of Pathology Ruhr University Bochum Germany; ^6^ Department of Visceral and General Surgery St. Josef Hospital Dortmund Germany; ^7^ Department of Visceral and General Surgery University Hospital Knappschaftskrankenhaus, Ruhr‐University Bochum Germany; ^8^ Institute of Medical Genetics and Applied Genomics University of Tübingen Germany

**Keywords:** CRC, EGFR, MEK, PDX, resistance, targeted therapy

## Abstract

Although approximately half of all metastatic colorectal cancers (mCRCs) harbour mutations in *KRAS* or *NRAS*, hardly any progress has been made regarding targeted treatment for this group over the last few years. Here, we investigated the efficacy of vertical inhibition of the RAS‐pathway by targeting epidermal growth factor receptor (EGFR) and mitogen‐activated protein kinase kinase (MEK) in patient‐derived xenograft (PDX) tumours with primary *KRAS* mutation. In total, 19 different PDX models comprising 127 tumours were tested. Responses were evaluated according to baseline tumour volume changes and graded as partial response (PR; ≤ − 30%), stable disease (SD; between −30% and +20%) or progressive disease (PD; ≥ + 20%). Vertical inhibition with trametinib and cetuximab induced SD or PR in 74% of analysed models, compared to 24% by monotherapy with trametinib. In cases of PR by vertical inhibition (47%), responses were lasting (as long as day 137), with a low incidence of secondary resistance (SR). Molecular analyses revealed that primary and SR was driven by transcriptional reprogramming activating the RAS pathway in a substantial fraction of tumours. Together, these preclinical data strongly support the translation of this combination therapy into clinical trials for CRC patients.

## Graphical abstract

Colorectal cancers with *RAS* mutations are resistant to epidermal growth factor receptor (EGFR) antibodies. Here, we demonstrate the efficacy of inhibiting EGFR and mitogen‐activated protein kinase kinase in 19 patient‐derived xenograft models with primary *KRAS* mutation. In responding tumours, we observed long‐term efficacy and only a few secondary resistant tumours. Resistance was mainly driven by transcriptional reprogramming, (re‐) activating the RAS pathway.

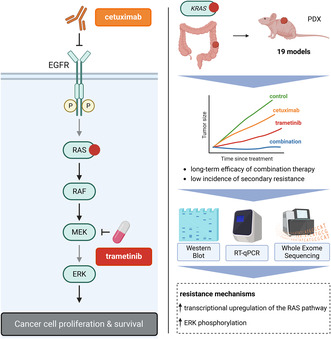

**Abbreviations**
AKT: protein kinase B
BRAF: v‐Raf murine sarcoma viral oncogene homolog B
CMS: consensus molecular subgroups
CR: complete response
CRC: colorectal cancer
DMSO: Dimethyl sulfoxide
EGFR: Epidermal growth factor receptor
ERK: extracellular signal‐regulated kinase
GSEA: gene set enrichment analysis
KRAS: Kirsten rat sarcoma viral oncogene homolog
mCRC: metastatic colorectal cancer
MEK: Mitogen‐activated protein kinase kinase
MSI‐high: high microsatellite instability
PD: progressive disease
PDX: patient‐derived xenografts
PR: partial response
SD: stable disease
SR: secondary resistance/resistant
STAT3: Signal transducer and activator of transcription 3

## 1 Introduction

Despite the successful implementation of surveillance measures, colorectal cancer (CRC) remains the second leading cause of cancer‐related deaths worldwide [1]. CRC is curable in localized stages, but metastatic CRC (mCRC) mostly has a dismal prognosis. Apart from microsatellite instable mCRC, where immune checkpoint inhibitors now are standard of care, treatment of mCRC consists of 5‐FU based chemotherapy [2, 3]. Over the last years, different targeted treatment options have been added, depending on genetic features and sidedness of the primary tumour. For patients without activating mutations in the downstream effectors of the epidermal growth factor receptor (EGFR) pathway and a left‐sided tumour [4], addition of an anti‐EGFR antibody to chemotherapy has shown promising results with median overall survival (OS) of 30–33 months [5, 6]. However, anti‐EGFR targeted treatments are ineffective in tumours with activating mutations in *KRAS, NRAS* and *BRAF*. A high fraction of CRC (48%) harbour mutations in *KRAS* or *NRAS* as main drivers, and another approximately 8% of CRC have got *BRAF* mutations [7]. Taking sidedness into account, only 20%–30% of patients qualify for a potential treatment with anti‐EGFR antibodies. For the large group of *RAS* mutated tumours, chemotherapy is often combined with anti‐VEGFR antibodies, but this combination‐treatment could not reach the same OS rates as anti‐EGFR‐targeted therapy in clinical trials [8].

An additional problem is that after approximately 10–12 months of treatment with cetuximab or panitumumab virtually all anti‐EGFR‐treated mCRC acquire resistance. One reason for this secondary resistance (SR) are emerging single nucleotide variants often in *RAS* genes [9]. We and others have recently shown that in addition to the selection of driver gene mutations transcriptional reprogramming may account for a large fraction of acquired resistance development [10, 11]. In a model with clonal selection of *KRAS* G12V and G12C mutations, we were able to effectively re‐induce growth inhibition by combining the anti‐EGFR‐treatment with a mitogen‐activated protein kinase kinase (MEK) inhibitor [10]. Next to these preclinical data, the principle of vertical inhibition has shown efficacy in clinical trials in other tumour entities, such as melanoma [12], and also in the BEACON trial for CRC. Here, combined BRAF and EGFR inhibition improved the median overall survival (OS) by 40% in patients with *BRAF* mutated mCRC, compared to standard chemotherapy by an exclusively targeted therapy approach [13].

The promising data from our own preclinical *in vivo* data and the BEACON CRC trial led us to the hypothesis that vertical inhibition of EGFR and its downstream effector MEK could be a valid approach to improve the management of patients with *RAS* mutated CRC. This has been tested so far mostly in *in vitro* experiments [14, 15, 16].

We therefore designed a preclinical patient‐derived xenografts (PDX) mouse model trial. First, we used the combination of anti‐EGFR antibodies and MEK inhibitors to assess the response of the combination treatment versus monotherapies. Second, we assessed the phosphorylation of extracellular signal‐regulated kinase (ERK) and the expression of DUSP6 to demonstrate the treatment to be on target. Finally, we analysed the occurrence of SR under vertical inhibition and assessed mutational and transcriptional changes to examine underlying mechanisms.

Assessing 19 PDX tumour models, the combination of cetuximab and trametinib (i) yielded a high therapeutic efficacy, reaching disease control [either partial response (PR) or stable disease (SD)] in 14 of the 19 models (74%) tested, (ii) was on target concerning ERK and DUSP6, and (iii) rarely led to the development of SR in models with PR. In case of SR under the combination therapy, transcriptional reactivation of the RAS pathway is a potential mechanism to drive resistance.

## 2 Materials and methods

### 2.1 Patients and tissue

Tumour samples from patients with previously confirmed CRC were collected during surgery at two clinical centers from Ruhr‐University, Bochum. Written informed consent was obtained from all patients prior to surgery. The tissue collection was performed according to a protocol approved by the Ethics Committee of the Ruhr‐University Bochum (registry nos. 3841‐10, and 16‐5792). All experimental protocols followed the declaration of Helsinki. Tissue samples described in this work were collected from May 2012 until July 2015.

### 2.2 Generation of patient‐derived xenografts (PDX) treatment cohorts

Generation of the PDX cohort has been described in detail in our previous work [10]. In detail, animal experiments and care were in accordance with the guidelines of institutional authorities and approved by local authorities (no.: 84‐02.04.2015.A135, Landesamt für Natur, Umwelt und Verbraucherschutz, Northrhine‐Westphalia). Briefly, animal experiments were conducted at the central animal facility of the medical faculty at Ruhr‐University Bochum. Animals were housed in Individually Ventilated Cages (Temperature: 21 °C, humidity 55%, day and night cylces: 14 : 10 h, sterile litter, food and water *ad libitum*). Handling of animals was carried out under laminar airflow for prevention of infections. During treatment, animals were scored daily for any signs of duress, including body weight, appearance, and spontaneous behavior. From a total of 158 CRC PDX models, 19 models with *KRAS* exon 2 mutations were randomly selected for therapy testing (Table 1). To establish treatment cohorts, tumour pieces (1–2 mm) from early passage PDXs (≤ F6 generation) were soaked in undiluted matrigel (Becton Dickinson, Le Pont de Claix, France) for 15–30 min. and subsequently implanted subcutaneously onto 5‐ to 10‐week‐old female mice (NMRI‐Foxn1nu/Foxn1nu, Janvier, St Berthevin Cedex, France) at two sites (scapular region) using as many as 4 pieces per site. Tumours were allowed to grow to a size of 200 mm^3^, at which time mice were randomized in the treatment and control groups with five to six mice in each group. Tumour volumes were estimated from 2‐dimensional tumour measurements by bi‐weekly caliper measurements using the following formula: Tumour volume (mm^3^) = [length (mm) × width (mm)^2^]/2. Growth curves were established by determining mean tumour volumes at different time points relative to the mean tumour volume at treatment start. Treatment response was evaluated for each PDX model on day 28. Complete response (CR) was defined as an undetectable tumour by macroscopical inspection and PR by at least a 30% reduction in mean tumour volume compared to the mean tumour volume at the start of treatment. Progressive disease (PD) was defined as a more than 20% increase in mean tumour volume determined at least at two consecutive time points compared to the tumour volume at the beginning of treatment. All other measurements were defined as SD. Acquired or SR development was assessed based on individual tumour growth curves. Individual tumours which responded with SD or PR during the initial 28 days treatment period but progressed thereafter (20% increase in mean tumour volume compared to the mean tumour volume at the start of treatment) were considered SR. Throughout the manuscript, the term “(PDX) model” refers to all tumours derived from an individual patient's tumour, whereas the term “tumour” refers to an individual tumour within a PDX model.

**Table 1:** Summary of PDX tumours. n.d., no data.

Tumour	UICC	KRAS	Response to cetuximab and trametinib		Response to trametinib	
			Day 28	Day 59	Day 28	Day 59
BoC51	I	G12D	PD	PD		
BoC64	IV	G12D	PD			
BoC109	IIIC	G13D	PD			
BoC117	IVA	G12D	PD	PD	PD	PD
BoC122	IIIB	G12D	PD	PD	PD	PD
BoC2	IV	G12D	SD	SD	PD	PD
BoC19	IIA	A146T	SD			
BoC56	IIA	G12C	SD	SD		
BoC78	IIA	G12D	SD	SD		
BoC136	n.d.	G12V	SD	SD	PD	PD
BoC9	IV	G12D	PR	PR		
BoC14	IIA	A146T	PR	PR		
BoC46	IIA	G13D	PR			
BoC47	IIIB	G12D	PR	PR	SD	SR
BoC80	IB	G12V	PR	PR		
BoC105	IIIB	G12V	PR	PR	SD	SR
BoC130	IIIC	G12D	PR	PR		
BoC137	IVB	G12D	PR	PR	PD	PD
BoC147	IIIB	G12A	PR	PR	SD	SD

### 2.3 Treatment of PDX

Eight models (BoC2, BoC47, BoC105, BoC117, BoC122, BoC136, BoC137, and BoC147) were assigned to treatment with cetuximab and trametinib, both as monotherapy and combination therapy. Another 11 models were treated with the combination regimen, only (BoC9, BoC14, BoC19, BoC46, BoC51, BoC56, BoC64, BoC78, BoC80, BoC109, BoC130). Two models (BoC105 and BoC147) were used to compare the efficacy of different combinations of MEK inhibitors (binimetinib, cobimetinib, or trametinib) and anti‐EGFR antibodies (panitumumab or cetuximab). Mice were treated with cetuximab (25 mg·kg^−1^, twice weekly, i.p., Merck, Darmstadt, Germany) or panitumumab (8 mg·kg^−1^, twice weekly, i.p., Amgen, Munich, Germany), binimetinib (6 mg·kg^−1^, five subsequent days per week, p.o., Hycultec, Beutelsbach, Germany), cobimetinib (10 mg·kg^−1^, daily, p.o., Hycultec), or trametinib (0.5 mg·kg^−1^, five subsequent days per week, p.o., Hycultec). Treatment was initiated at 200 (+/− 30) mm^3^. Controls remained untreated.

### 2.4 Intermittent long‐term treatment

In order to induce SR, four PDX models responding with PR to the combination therapy were subjected to intermittent long‐term treatment as has been described previously [10]. If tumours reached PR until day 59, the treatment was paused until the tumour regrew to a volume of 200 mm^3^ before treatment was re‐initiated. If PR or SD was achieved again for at least 30 days, the treatment was paused again and re‐initiated if the tumour roughly doubled its volume relative to its volume at the end of the last treatment cycle. Two to three treatment cycles were performed this way.

### 2.5 Immunoblot analysis

Total protein was extracted from tumours treated for five subsequent days with trametinib and two doses of cetuximab (days 1 and 4) or at the end of treatment (EOT) in case of primary or secondary resistant (SR) tumours, as well as from untreated controls. Three hours after the last application of trametinib, tumours were explanted and immediately shock‐frozen in liquid nitrogen. All samples were stored at −80 °C. Total cellular proteins were extracted by solubilizing the cells in lysis buffer 17 (R&D Systems, Minneapolis, MN, USA) in the presence of a mixture of protease (cOmplete Mini, Roche, Basel, Switzerland) and phosphatase inhibitor cocktail 2 (Sigma‐Aldrich, Taufkirchen, Germany), and the lysates were subsequently sonicated, and cellular debris was removed by centrifugation. Western blot detection was performed with an enhanced chemiluminescence system (BioRad, Hercules, CA, USA), SuperSignal^®^ West Pico Chemiluminescent Substrate (Thermo Scientific, Schwerte, Germany), and peroxidase conjugated secondary antibodies (Santa Cruz Biotechnology, Heidelberg, Germany or dianova, Hamburg, Germany). The following primary antibodies were used for Western blotting (all from Cell Signaling Technology, Danvers, MA, USA): anti‐p44/42 ERK (1 : 3500), anti‐phospho‐p44/42 ERK (Thr202/Tyr204; 1 : 1000); anti‐AKT (1 : 5000), anti‐phospho AKT (Ser473; 1 : 2000); anti‐phospho AKT (Thr308; 1 : 1000); anti‐S6 ribosomal protein (1 : 5000), anti‐phospho S6 Ribosomal protein (Ser235/236; 1 : 1000); anti‐beta‐actin (1 : 1000); anti‐Stat3 (1 : 2000), anti‐phospho Stat3 (Tyr705; 1 : 2000); anti‐GAPDH (1 : 40 000).

image lab 5.0 (BioRad) was used to quantify the amount of protein. Total and phosphorylated protein levels were normalized using the ratio of beta‐actin/GAPDH in treated and control tumours. Using beta‐actin/GAPDH as baseline, the relative fold change was then calculated between control and treated tumours.

### 2.6 Targeted next generation sequencing

All tumours were analysed by targeted sequencing, as has been described before [10]. Briefly, 250 ng genomic DNA was used per sample to produce sequencing libraries with the TruSeq Amplicon Cancer Panel (Illumina, San Diego, CA, USA) according to the manufacturer's protocol. The chosen panel covered 48 cancer‐related genes with 212 amplicons, which were simultaneously amplified in a single tube reaction (Fig. S1, Table S1). Briefly, the regions of interest were enriched by hybridizing sequence‐specific oligonucleotides to the genomic DNA followed by ligation extension of the bounded oligos. The marked regions were further amplified by PCR with primers containing index barcodes for sample multiplexing. Finally, libraries were normalized by bead normalization prior to sequencing. Pooled libraries were sequenced on a MiSeq instrument (Illumina) using 2 × 150 bp paired‐end reads. For data processing, fastq files were analysed with the nextgene V2.3.4 (SoftGenetics, State College, PA, USA) software. For variant calling raw reads were aligned to the human hg 19 assembly and primer sequences were soft‐clipped prior to variant calling. Variants with a minor AF of ≥ 5% within the coding region and a minimum coverage of 10 variant reads were considered as alteration and visually confirmed with nextgene. In case of “likely pathogenic” or “pathogenic” variants in ClinVar, we considered these alterations in our analysis.

### 2.7 Quantitative real time PCR (qRT‐PCR)

Quantitative real time PCR was conducted similaraly to a previous work [10]. cDNA was synthesized using 2 μg of total RNA, oligo(dT)18 primers and M‐MLV reverse transcriptase (Promega, Madison, WI, USA) following the manufacturer's protocol and diluted to a final volume of 50 μL with 1× first strand buffer. Intron spanning primer sets for qRT‐PCR were designed using primer express 2.0 software (Applied Biosystems, Waltham, MA, USA) (DUSP6‐1584‐s: ACAAGCAAATCCCCATCTCG, DUSP6‐1784‐as: TGTCATAGGCATCGTTCATCG). qRT‐PCR was performed using a SYBR Green I reaction mixture containing 75 mm Tris–HCl (pH 8.8), 20 mm ammonium sulfate, 0.01% (v/v) Tween 20, 2 mm magnesium chloride (all Sigma‐Aldrich), 1 μL of a 600‐fold dilution of SYBR Green I (BioWhittaker, Apen, Germany), 2.5 U Taq polymerase (NEB, Ipswich, MA, USA), 0.2 mm dNTP (Promega) and 0.2 μm of forward and reverse primer in a final reaction volume of 20 μL. Reactions were run on a CFX Connect Real Time System (Bio‐Rad). The cycling conditions consisted of 3 min initial denaturation at 94 °C and 40 cycles of 94 °C for 30 s, 60 °C for 30 s, 72 °C for 30 s and 80 °C for 5 s. Fluorescence was measured at the last step of each cycle. Melting curves were obtained after each PCR run and showed single PCR products. cDNAs were run in triplicate, non‐RT (without reverse transcriptase), and no‐template controls were run in duplicates. Expression ratios were calculated as described by M. Pfaffl [17] using the geometric mean expression of the housekeeping gene GUSB (GUSB‐1430‐s: GGTGCGTAGGGACAAGAACC, GUSB‐1550‐as: CAAGGATTTGGTGTGAGCGAT) to normalize the expression data for the gene of interest.

### 2.8 Whole exome sequencing

Whole exome sequencing was performed using 100 ng of genomic DNA. Enzymatic fragmentation, A‐tailing, adapter ligation and amplification was performed using the Twist Library Preparation Kit Enzymatic Fragmentation EF2.0 (Twist Bioscience, South San Francisco, USA). Pools of eight amplified libraries using 200 ng of each were used for targeted capture with an extended version of the Twist Human Core Exome enrichment kit covering over 50 Mb of regions of interest. The DNA was amplified by PCR, and quality and quantity of the sequencing library were assessed using the Qubit fluorometer (Thermo Fisher Scientific, Waltham, USA) and 2200 TapeStation (Agilent Technologies, Santa Clara, USA), respectively. The library was then sequenced on the Illumina NovaSeq 6000 platform (Illumina, San Diego, USA) using 2 × 100 bp paired‐end reads.

Raw data QC and processing was performed using the ngs‐bits toolkit (version 2023_03) [18] and the megSAP pipeline (https://github.com/imgag/megSAP, version 2023_03). Briefly, sequencing reads were aligned to a combination of the human and mouse reference genomes (GRCh38 and GRCm38) by BWA‐MEM2 [19], variants were called using VarScan [20] and annotated with VEP [21]. To obtain high‐confidence results only variants located on human chromosomes and the exome enrichment target region were considered. Known variants with a population allele frequency above 0.1% in gnomAD (overall population frequency) or above 0.1% in any subpopulation were excluded. Variants below 5% allele frequency or with modifier variant type according to the Sequence Ontology classification were discarded as well. To assess tumour relevance, remaining variants were annotation with information from the COSMIC database and the Network of Cancer Genes (NCG) [22]. Only mutations ranked by the Cancer Mutation Census (https://cancer.sanger.ac.uk/cmc/help) between tier 1 and 3 were further considered. Exome sequencing has been deposited into the NCBI BioProject database under the BioProject ID PRJNA 988418 (Reviewer Link: https://dataview.ncbi.nlm.nih.gov/object/PRJNA988418?reviewer=ehlehc7iirecmffhm6cgisj2c8).

### 2.9 Gene expression analyses and data processing

Gene expression analysis and data precessing was carried out as has been described before [10]. An amount of 100 ng of every total RNA sample was hybridized to Agilent whole‐genome expression microarrays (Human GE 4x44K, v2 G4845A, AMADID 026652, Agilent Technologies). RNA labeling, hybridization, and washing were carried out according to the manufacturer's instructions. Images of hybridized microarrays were acquired with a DNA microarray scanner (Agilent G2505B) and features were extracted using the agilent Feature Extraction image analysis software (AFE) version A.10.7.3.1 with default protocols and settings. The AFE algorithm generates a single intensity measure for each feature, referred to as the total gene signal (TGS), which was used for further data analyses using the r package limma version 3.50. The normalization of the data was performed by the quantile method that is implemented in the limma package [23]. TGS were normalized by the quantile method. Subsequently, the data was filtered based on the normalized expression values, using the moderated t‐test and multiplicity correction was included to control the false discovery rate (FDR) at 0.05% [24]. The gene expression data from our study have been deposited in the NCBI's Gene Expression Omnibus (GEO) database (accession number GSE236078; https://www.ncbi.nlm.nih.gov/geo/query/acc.cgi?acc=GSE236078).

### 2.10 Gene set enrichment analyses (GSEA)

Gene set enrichment analyses software [25] (V4.3.2) was provided by the Broad Institute of the Massachusetts Institute of Technology and Harvard University (http://www.broad.mit.edu/gsea/). Compared to a previous work [10], this time the hallmark gene sets (V2023.1) were used with default parameters of the GSEA software package; gene set permutation was used. Gene sets with FDR *q*‐val ≤ 0.05 were considered appropriate.

### 2.11 Statistical analysis

The statistical analyses and plotting of data were carried out using r 4.1.2. For growth curves, the mean per model and treatment arm was calculated and plotted. The two‐sided unpaired Student's *t* test was used to evaluate the statistical significance of differences between treated groups. A *P*‐value < 0.05 was regarded as statistically significant. Means and standard error of the mean are shown. Waterfall plots depict either the last value before end of the experiment or on day 59. For axis breaks, we used ggbreak v0.1.1 [26].

## 3 Results

### 3.1 *In vivo* pilot efficacy tests of different MEK inhibitors in combination with anti‐EGFR antibodies

We first compared the efficacy of three MEK inhibitors (trametinib, cobimetinib, and binimetinib) combined with anti‐EGFR antibodies in two randomly chosen *KRAS* mutated CRC PDX models *in vivo*, which have previously shown effective *in vitro*. Selumetinib was not included because it was of reduced benefit in comparative preclinical experiments and minimal efficacy in a phase I clinical trial [27, 28, 29]. All tested combinations led to tumour growth inhibition but only the combination of trametinib and cetuximab was able to induce PR in both models (Fig. S2). Therefore, we chose cetuximab and trametinib for the following *in vivo* studies.

### 3.2 Cetuximab and trametinib induced sustained tumour growth inhibition in KRAS mutated CRC PDX

**Fig. 1:**
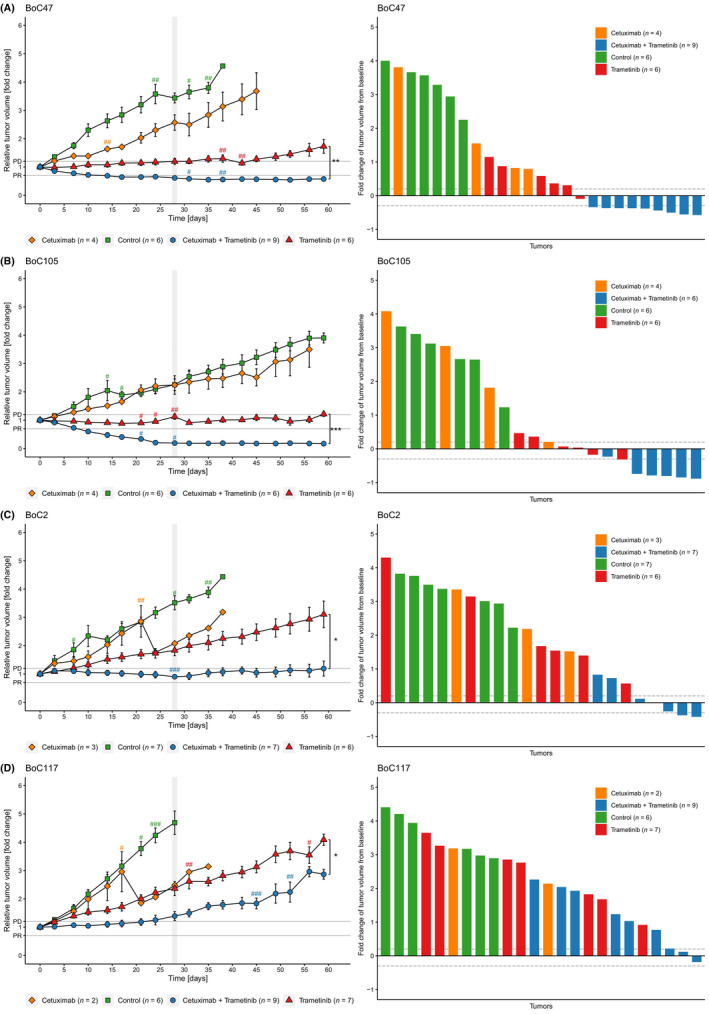
Growth curves and waterfall plots of representative PDX models. Relative growth curves are derived from mean values ± SEM (error bars). Each ^#^ represents a tumour that was taken out of the treatment cohort at the indicated time point either because the tumour reached the maximum size criteria or due to health issues of the animal. The combination treatment with cetuximab and trametinib either showed PR (A and B), stable disease (C) or PD (D). Vertical gray bars indicate end of the primary observational period of 28 days. Waterfall plots show the response at the end of observation for each individual tumour (maximum observational period: until day 59). Each bar represents one tumour. Dotted lines indicate the cut‐off values for PD and PR. *, *P* < 0.05; **, *P* < 0.01; ***, *P* < 0.001, determined by Student's *t*‐test.

In a first set of therapy tests, eight models were treated with cetuximab and trametinib, either in combination or as monotherapy. Primary response was evaluated 28 days after treatment initiation comparing mean tumour volumes on day 28 with the mean tumour volume at the start of treatment for all treatment arms (Fig. 1A–D, Figs S3A–D, S4A–E, S5A–C and S6A–C, left column, gray bar indicating day 28). Furthermore, waterfall plots show relative tumour volumes at the EOT or latest on day 59, respectively (Fig. 1A–D, Figs S3A–D, S4A–E and S5A–C, right column). Notably, in some cases individual tumours of the same model had a different response than the mean response of the entire cohort to a specific treatment. As expected, all cetuximab treated models progressed (Fig. 1A–D, Fig. S3A–D). Compared to that, trametinib monotherapy was only able to induce disease control (i.e. SD) in three models (BoC47, BoC105, BoC147). However, the remaining five models (BoC2, BoC117, BoC122, BoC136, BoC137) were primary resistant to trametinib. As the monotherapies did not achieve a substantial tumour growth inhibition, we omitted the monotherapy in the subsequently tested 11 PDX models.

The combination therapy proved to be most effective, with PR in 9 of 19 models tested (BoC9, BoC14, BoC46, BoC47, BoC80, BoC105, BoC130, BoC137, BoC147) and SD in another five models (BoC2, BoC19, BoC56, BoC78, BoC136). In BoC51, BoC64, BoC109, BoC117 and BoC122, we achieved growth control for less than 28 days and therefore considered them primary resistant (Figs 4, 5, 6). Worth mentioning, in models with primary resistance, progression became only evident after an initial phase of growth control of approximately 15–21 days.

**Fig. 2:**
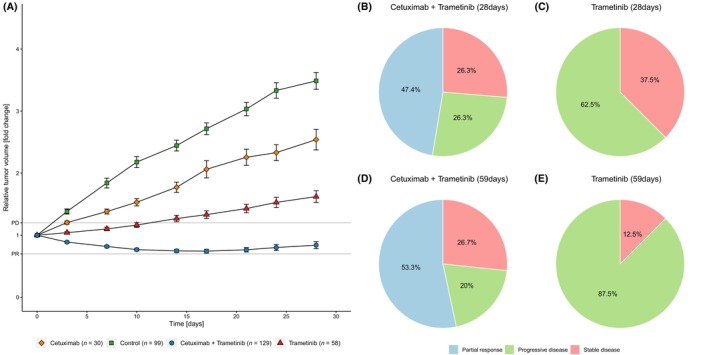
Summary of all treated tumours and response data. Growth curves of all tumours summarizing all 19 cohorts. Data shown until day 28 (A) (error bars indicating SEM). Response patterns of cetuximab and trametinib on day 28 based on 19 models (B) and day 59 based on 15 models (D), and for monotherapy with trametinib on day 28 (C) and day 59 (E), both with 8 models.

**Fig. 3:**
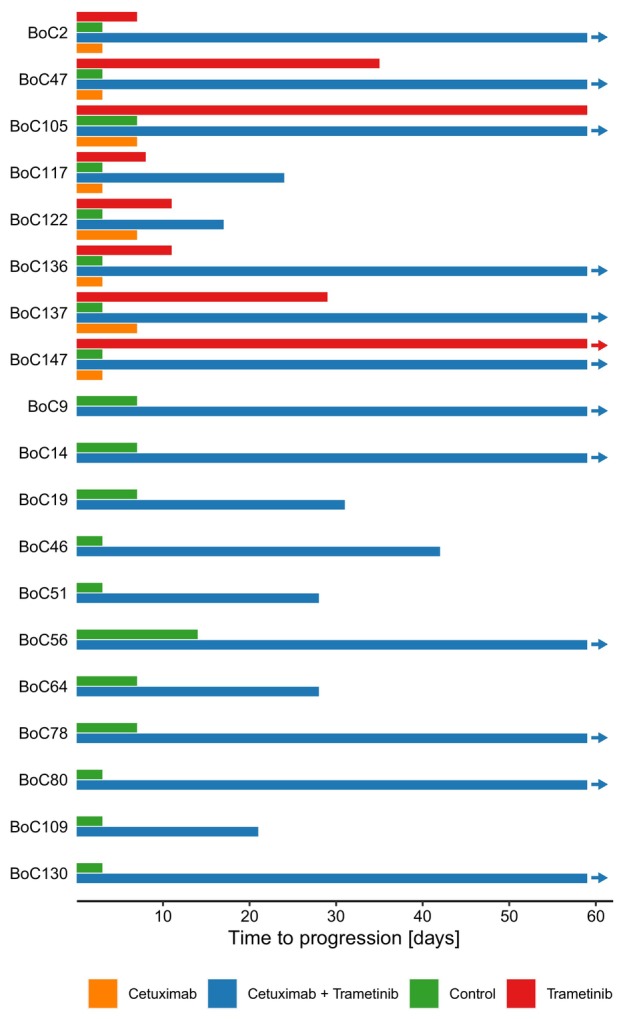
Time to progression of PDX models. Time to progression depicted as mean time (days) for tumour models meeting progression criteria (volume gain of at least 20%). In 12/19 models (63%) vertical inhibition showed responses beyond day 59 compared to 1/8 models with MEK‐Inhibition alone (13%). The end of each bar represents the mean value of treated tumours meeting criteria for PD. Arrows indicate tumours still responding at the EOT.

A total of 316 individual tumours in 19 PDX models were treated for the primary observational period (day 28) of which 129 received the combination therapy, 20 were treated with cetuximab only, and 58 with trametinib only, respectively (99 controls) (Fig. 2A). Summarizing mean relative tumour volume changes over all models for each treatment arm (Fig. 2A), we observed some growth inhibition with cetuximab monotherapy, and a somewhat more pronounced response with MEK inhibition. Nevertheless, the overall response for both treatment arms was PD at day 28 (Fig. 2A). However, vertical inhibition with cetuximab and trametinib led overall to disease control (Fig. 2A). Individual response patterns for each of the 19 models gave a more detailed picture: Combination treatment achieved PR in 9/19 models (47%), and SD in 5/19 models (26%). In 5/19 (26%) models treatment failed and therefore these models were considered primary resistant (Fig. 2B). In summary, the combination of cetuximab and trametinib could induce disease control (PR or SD) in 14/19 (74%) models (Figs 2B–E and 3). Importantly, this response pattern based on mean tumour volumes remained stable over the extended treatment period of 59 days for all models tested (Fig. 2D). Trametinib monotherapy reached within the primary observational period of 28 days a disease control rate of only 37.5% (Fig. 2C) and the fraction of tumours with PD increased over time to 87.5% in this cohort (Fig. 2E). In contrast, in 12/19 models (63%) with the combination therapy a lasting response was detected beyond day 59 (Fig. 3) compared to 1/8 models (12.5%) treated with trametinib, only (Figs 2E and 3).

Secondary resistance was defined as PD after disease control during the first 28 days of treatment. Assessing individual tumour growth curves, we noted that in 4 models (BoC2, BoC56, BoC78, and BoC136) altogether 6 of 25 treated tumours fulfilled the criteria for SR. Of note, SR tumours were only observed in models with a mean response pattern of SD at day 28 but never in models that showed PR as mean response at day 28 [in Fig. 1C (BoC2); Fig. S3C (BoC136) Fig. S5b (BoC56) and Fig. S5C (BoC78)]. Thus, a deeper, objective response was associated with a longer‐lasting response.

### 3.3 Intermittent long‐term treatment for the induction of secondary resistance

In an attempt to induce SR tumours in models responding to the combination with PR we implemented a protocol with treatment pauses in tumours responding to the combination therapy and treatment re‐initiation once a tumour showed clear signs of regrowth in analogy to our previous work [10].

**Fig. 4:**
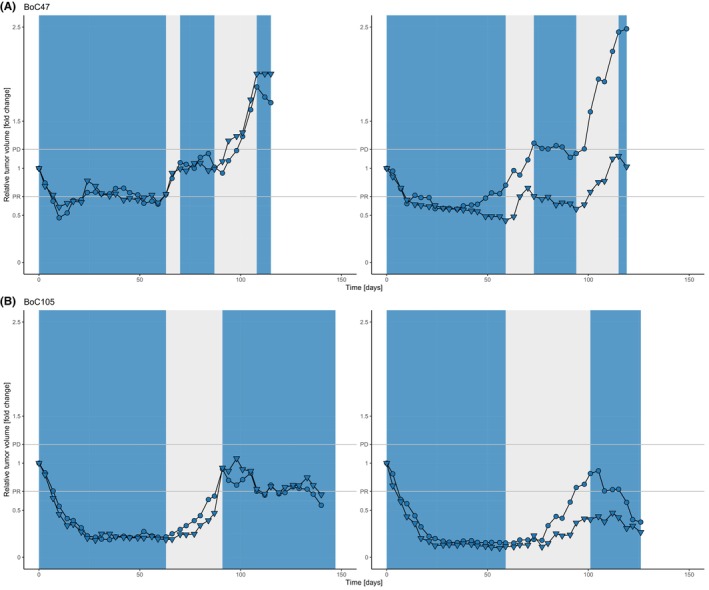
Low incidence of SR upon intermittent treatment cycles. Shown are data from two representative examples of the two models BoC47 (A) and BoC105 (B) (8 tumours). Growth curves are derived from pairs of tumours growing on an individual mouse. In models responding until day 59, treatment was paused until tumour showed clear signs of re‐growth. Treatment was re‐initiated and in case of disease control for at least 30 days, the treatment was paused again. A third treatment cycle was started upon re‐growth. The phases of treatment pause are highlighted in gray, treatment periods in blue. Only one tumour of the BoC47 PDX model showed SR (A, right panel, top growth curve).

Thirteen individual tumours from four models (with two to four tumours per model) were monitored under this treatment schedule (Fig. 4, Fig. S7). Two models clearly responded again after re‐induction of treatment and therefore SR was not observed during the follow‐up time with a cumulative time under treatment (including treatment pauses) ranging from 116 to 137 days **(**BoC105 Fig. 4B, BoC147 Fig. S7B). In two models (BoC137 and Boc47), one tumour each progressed in the third treatment cycle (Fig. S7A, right panel). Similarly, for BoC 147, one tumour did not show a clear response at the EOT. In addition, in one tumour each of the same models, no volume reduction was observed in the last treatment cycle. Nevertheless, in these tumours the combination still achieved SD. In summary, two of thirteen tumours (15%) showed clear signs of SR development after treatment re‐initiation compared to the initial treatment phase whereas the remaining 11 tumours showed disease control.

### 3.4 Combination therapy suppresses phosphorylation of ERK and S6

**Fig. 5:**
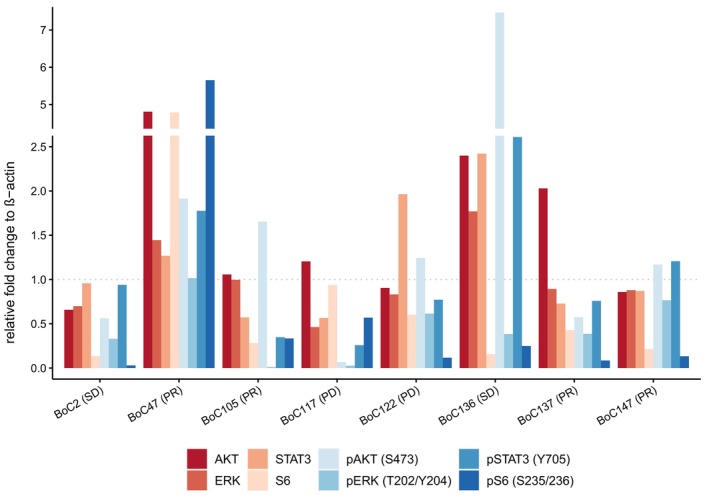
Western blots of combination treated tumours. Protein intensity was measured by image lab software. Presented values display the protein levels normalized by beta‐actin and set in relation to control tumours. Upper axis shows treatment response to combined therapy with cetuximab and trametinib. All Western Blot experiments were replicated at least once [Number (*n*) of replicates for BoC2, BoC117, BoC122, BoC147: *n* = 4; BoC47, BoC105, BoC136: *n* = 3; BoC137: *n* = 2].

Western blot analysis was performed to investigate downstream phosphorylation of direct effectors of the MAPK pathway (ERK and S6) as well as potential bypass pathways (AKT and STAT3**).** The samples for western blot analysis were harvested on day 5 of the combination treatment with cetuximab and trametinib (5dCT). The reason for this approach was to clarifiy, if the activitiy of the MEK–ERK pathway was already lost or still maintained in this early treatment phase of combination therapy. Responses are depicted in Fig. 5 as bar graphs and the corresponding western blots are shown in Figs S8 and S9.

Compared to control tumours, all but one analysed tumour (BoC47) showed suppression of ERK and S6 phosphorylation on day five (Fig. 5, Figs S8 and S9C), independent of their treatment response. This is in line with the growth behavior of all tumours, where even primary resistant models showed an initial phase of growth control for more than 5 days. Some tumours showed an early activation of potential bypass‐pathways as through increased phosphorylation of AKT or STAT3. A marked rise in phosphorylated AKT (pAKT) was observed only in BoC136 (Fig. 5, Fig. S8) and a somewhat moderate (between 1.5‐ and 2‐fold) increase in BoC47 and BoC105 (Fig. 5, Fig. S8). The two tumours with the strongest rise in pAKT also showed an increase in STAT3 phosphorylation. However, none of these tumours developed SR during the follow‐up of 59 days.

Furthermore, for a subset of primary (BoC64, BoC109, BoC117, BoC122) and SR PDX models (BoC2, BoC56) Western blot analysis was performed (Fig. S9A,B). Except for primary resistant tumour BoC117 (Fig. S9A), all analysed tumours showed an increase of phosphorylated ERK (pERK) at the EOT compared to day 5 irrespective of being primary or SR. However, the level of pERK without any treatment was only exceeded in one primary resistant model (BoC64, Fig. S9A). The levels of pAKT varied over time points, phosphorylation site, and tumours. Some models showed a rise of pAKT after 5 days with a subsequent decline to the EOT (primary resistant BoC117, Fig. S9A). Others showed a consecutive drop of pAKT levels (SR BoC2) or an initial decline at day 5 with a consecutive gain in pAKT levels [primary resistant BoC109, SR BoC56 for pAKT(T308)] (Fig. S9). In summary, a clear pattern regarding pAKT could not be detected.

### 3.5 Reactivation of the RAS pathway in primary and secondary resistance

**Fig. 6:**
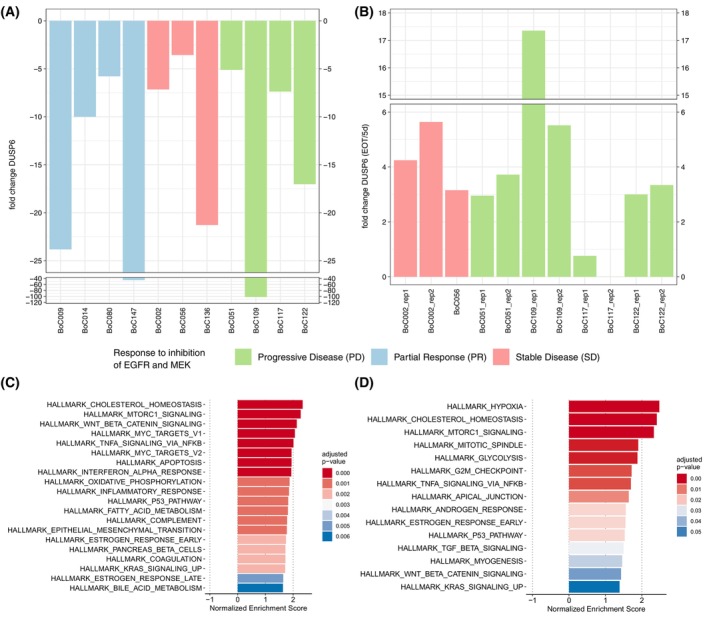
Expression of DUSP6 and GSEA. Expression of DUSP6 after 5 days of treatment (A) and end‐of‐treatment compared to 5 days of treatment (B); all in comparison to control tumours. Data was derived from cDNAs run in triplicate during qRT‐PCR: GSEA for primary (C) and SR (D) models. GSEA analysis for primary resistant models was performed on gene expression data derived from 8 resistant compared to the corresponding 8 sensitive (treated for 5 days with cetuximab and trametinib) tumours and for SR models from 4 SR and sensitive tumours each.

Dual specificity phosphatase 6 (DUSP6) is central in the negative feedback regulation of the KRAS signaling pathway and its expression level is used as a surrogate marker for RAS pathway activity. We investigated the expression of *DUSP6* by RT‐qPCR in individual tumours from 11 tumour models after 5 days and at the EOT. Like the ERK phosphorylation data shown above, all tumours tested showed reduced *DUSP6* expression levels on day five of treatment compared to untreated control tumours. This included one tumour of the BoC14 model, which lacked ERK phosphorylation reduction upon treatment (Fig. 6A).

Upon primary or SR development, we observed in all but one tumour a relative increase in ERK phosphorylation levels (Fig. S9) and *DUSP6* expression (Fig. 6B). The lack of reactivation of ERK phosphorylation in one SR tumour (BoC117) (Fig. S9A) was accompanied with a comparable low rise in *DUSP6* expression (Fig. 6B). These data indicate that reactivation of the RAS pathway is important in the majority of tumours in our cohort for resistance development to MEK inhibition. Next, we asked, if our initial targeted sequencing to select the PDX models may have missed mutations potentially driving primary resistance. Therefore, we performed exome sequencing for one primary resistant tumour each, from four different PDX models (BoC51, BoC109, BoC117, and BoC122). Additional pathogenic mutations were not detected in any tumour except for the microsatellite instable BoC109 tumour, which harbored a heterozygous mutation inactivating mutation in the *NF1* gene (allele frequency of 0.46) (Fig. S10, Table S2). Similarly, we analysed a SR tumour each of two models (BoC2 and BoC56) by exome sequencing. In neither model we could identify any mutation selected or arising in the resistant tumour in comparison to the untreated control tumour beyond the known pre‐treatment *KRAS* mutation (Fig. S10, Table S2).

Further, we sought to use transcriptome analyses to gain insight if transcriptional reprogramming may explain the reactivation of the RAS pathway in the resistant tumours as suggested by our *DUSP6* analyses. From the same set of primary resistant PDX models assessed via exome sequencing we analysed two tumours each treated for 5 days with the combination (while the tumour was still responding to the treatment) and two primary resistant tumours each (harvested at the end of the combination treatment, except for BoC56, where only one primary resistant tumour was available for analysis), by standard gene expression array analyses. in agreement with the *DUSP6* data, gene set enrichment analysis (GSEA) using the hallmark gene sets revealed that the “KRAS_SIGNALING_UP” gene set was among the significantly enriched sets in primary resistant tumours. Moreover, additional gene sets known to support cell proliferation such as the “MYC_TARGETS”, “TNFA_SIGNALING_VIA_NFKB”, and “MTORC_1_SIGNALING” sets were enriched (Fig. 6C). In SR models, the “KRAS_SIGNLING_UP”, “TNFA_SIGNALING_VIA_NFKB”, and “MTORC_1_SIGNALING” gene sets were also enriched, as well as additional gene sets known to be associated with oncogenic signaling such as “GLYCOLIYSIS”, and “HYPOXIA” (Fig. 6D). Frequently upregulated MAPK pathway genes in resistant tumours were apart from *DUSP6* different *FGFs*, *MYC*, *EPHA2*, and *FOS* (Table S3).

Finally, the consensus molecular subgroup (CMS) classification of 9 untreated control tumours (including the four primary resistant and two SR tumours shown above) was done with the recently introduced CMScaller, which was somewhat optimized for the analysis of pre‐clinical models such as PDX models [30]. This did not reveal any CMS class linked to primary or SR (Table S4). In agreement with what has previously been described, the *KRAS* mutated PDX models included were not strongly confined to a specific CMS class, albeit CMS1 was somewhat overrepresented with 4/9 models in this group [31]. This CMS1 class was reported to be enriched for microsatellite instable tumours [30, 31]. However, in our series, only BoC109 belongs to this group. Although we could not correlate the CMS classification with resistance patterns, our RT‐qPCR and transcriptomic data showed increasing *DUSP6* levels as a sign of resistance development over time and upregulation of, among others, the typical *KRAS* driven hallmark pathways.

## 4 Discussion

Over the last decade, systemic treatment of mCRC has hardly changed. Monoclonal anti‐EGFR antibodies in combination with chemotherapy were able to improve prognosis in a subgroup of patients with median OS rates greater than 30 months [6, 32]. As these treatments are only effective in left‐sided *RAS* and *BRAF* wildtype CRC, the majority of patients do not benefit from anti‐EGFR targeted therapy. In this case, anti‐VEGFR targeting agents are combined with similar chemotherapy backbones, but survival data are less promising compared to the *RAS* and *BRAF* wildtype group of patients [33].

In other molecular subgroups, new therapeutic strategies have provided promising results. Based on the BEACON CRC trial, the combination of the anti‐EGFR antibody cetuximab and the BRAF inhibitor encorafenib is now approved in *BRAF* mutated mCRC after progressing on first‐line chemotherapy. Compared to a standard polychemotherapy regimen (FOLFIRI), cetuximab and encorafenib proved to be superior in terms of overall survival by vertically inhibiting the RAS pathway. However, the addition of a MEK inhibitor as a triple targeting therapy did not offer an additional benefit [13]. Notably, the treatment concept of dual blockade to prevent feedback activation of the EGFR was initially demonstrated in xenograft experiments [34]. KRAS‐directed therapies are under development. More recently, an allosteric KRASG12C inhibitor, as well as a non‐covalent KRASG12D inhibitor have become available [35, 36]. While the KRASG12D inhibitor is still under preclinical investigation, the allosteric KRASG12C inhibitor, sotorasib, has proven to be successful and has been approved in lung cancer [37]. Unfortunately, data for CRC are less convincing and only a small fraction of CRC show a KRAS G12C variant [38]. The likely reason for the limited activity of the G12C inhibitors is alternative growth factor signals via upstream RTKs inducing a strong phospho‐ERK rebound via the activation of the wild‐type KRAS [39]. Similar to what was observed with BRAF inhibitors, the EGFR receptor seems to be the dominant resistance mediator, and vertical pathway inhibition to prevent adaptive feedback activation is critical to generate a sufficient response using KRAS inhibitors [40, 41]. Although clinical data for the G12D inhibitor are not yet available, the pre‐clinical xenograft data also hint towards the need for vertical inhibition for the induction of optimal tumour growth inhibition [36]. Despite the astounding improvement of prognosis regarding subgroups of patients with mCRC such as patients harboring tumours with microsatellite instability, to date no effective targeted treatment is available for the majority of *RAS*‐mutated CRC patients illustrating the urgent need for this patient group for novel treatment options [3, 42].

Previous data from Misale et al., as well as our own data, showed that tumours selecting *KRAS* mutations during acquired resistance development under anti‐EGFR therapy can be successfully treated by vertical inhibition of EGFR and MEK [9, 10, 43]. Similar findings were reported by Troiani et al. for a low number of primary *KRAS* mutated CRC cell lines combining cetuximab with the selective MEK1/2 inhibitor refametinib [15]. Surprisingly, in one phase I trial combining the MEK inhibitor selumetinib with cetuximab no objective response was observed [28]. These rather less convincing data may not only be caused by the heavy pretreatment of the patients but also by the selection of selumetinib, a MEK inhibitor with inferior activity in pre‐clinical experiments [27, 29, 44]. Therefore, we chose to evaluate vertical inhibition in a large cohort of PDX models combining anti‐EGFR antibody therapy with a more potent MEK inhibitor. Our pilot test of MEK inhibitors with higher potency compared to selumetinib and refametinib such as cobimetinib (MEK1 specific inhibitor), binimetinib (MEK1/2 inhibitor) or trametinib (MEK1/2 inhibitor) in combination with anti‐EGFR antibodies indicated that trametinib may be the MEK inhibitor with the best activity profile and was therefore used in our pre‐clinical study. This combination achieved disease control in 14/19 *KRAS* mutated CRC models (74%) with an excellent PR rate of 47%. In PDX models with a response pattern of SD, a subgroup of altogether 6 individual tumours developed SR (6/25, 24%). For PDX models initially showing PRs till day 28. Lasting tumour growth control for more than 100 days was achieved in the 85% of tumours tested by integrating treatment pauses between the treatment cycles which were adapted to the growth behavior of the tumour. This might hinder potentially resistant clones to outgrow and limit treatment‐related toxicities. However, in a subgroup of tumours tested with growth control the treatment response became somewhat attenuated reaching only SD. Extended intermittent treatment tests will be necessary, to determine if this has to be considered as an early sign of imminent SR development. From a translational perspective, this concept could be best compared to current re‐challenging strategies in mCRC regarding anti‐EGFR treatment [45]. Our findings confirm the long‐term efficacy of MEK and EGFR inhibition as first line therapy in PDX models with a low rate of rapid development of SR in the subgroup of patients with PR. As SR evolves in almost any clinical setting [46, 47, 48], preventing SR is crucial to induce and sustain lasting responses in patients. We expect, similar to findings in *BRAF* ^*V600E*^ mutated melanoma, that combining MEK and EGFR inhibition should also postpone SR and prolong survival of CRC patients [12, 49].

Western blot analyses at an early time point following treatment induction showed reduced phosphorylation of ERK in all but one tumour analysed, confirming successful suppression of the RAS–MEK–ERK pathway. This was corroborated by the reduction of *DUSP6* expression, a protein well‐known for its involvement in the negative feedback control of the activated RAS pathway. We also observed higher AKT phosphorylation levels in three of four tumours with PR. This is in contrast to a previous report [50], indicating that AKT activation is not necessarily inducing resistance to MEK inhibition and that the overall signaling context of the tumour cell determines whether or not resistance occurs. The availability of tumours developing primary or SR under the combination enabled us to demonstrate the recovery of phosphorylated ERK in virtually all primary and SR models over time (Fig. 5, Figs S8 and S9). Importantly, even in the primary resistant models, we observed an initial reduction of the pERK, which was recovered during resistance development (Fig. S9A). To identify a known mutation able to potentially drive resistance development beyond the *KRAS* mutation, an exome sequencing‐driven attempt failed in all but one of the tested primary or SR tumours. The heterozygous inactivating neurofibromin 1 (*NF1*) mutation discovered in the primary resistant tumour BoC109, is likely to contribute to a reduced sensitivity of the vertical inhibition by reducing the effect of cetuximab on RAS signaling. This is supported by data from the cetuximab‐resistant CRC cell line KM12C harboring a *NF1* truncating mutation with a similar allele frequency, in which restoration of the NF1 function reduced MEK and ERK phosphorylation as well as cell proliferation and increased cetuximab induced apoptosis [51]. Gene expression analyses, in turn, revealed in agreement with the observed *DUSP6* rise in the majority of resistant tumours, that re‐activation of the RAS pathway is critical for developing primary and SR (Fig. 6C,D). Furthermore, these data imply that similar to what has been described for SR *RAS* wild‐type CRCs treated with cetuximab monotherapy [10, 11], primary and SR towards the vertical inhibition tested herein may be driven by their inherent transcriptional plasticity in a substantial subgroup of tumours. Moreover, transcriptional reprogramming may compensate for the lack of mutations to reinstall the critical RAS pathway activity.

In this preclinical study, altogether 127 individual tumours were treated with the combination of trametinib and cetuximab. As both substances are already approved in other applications, we strongly recommend initiating a clinical trial in order to evaluate the concept of vertical inhibition in *RAS* mutated mCRC. In the current setting of rather unsatisfying results in mCRC regarding strategies directly targeting RAS as discussed above, vertical inhibition of the pathway might be the best chance of improving prognosis for these patients.

## 5 Conclusions

To the best of our knowledge, this is the largest PDX trial focusing on *KRAS*‐mutated CRC. Vertically targeting the RAS signaling pathway by inhibiting EGFR and MEK led to a sustained PR in 47% of analysed tumour models and tumour growth control in more than 70%. Furthermore, tumours showing an initial response did rarely develop SR to dual targeted treatment. In case of SR, transcriptional reprogramming might be an underlying mechanism. Our pre‐clinical *in vivo* data support that the combination of EGFR and MEK inhibition is a potent treatment option which should be tested in future clinical trials addressing *KRAS* mutated CRCs.

## Conflict of interest

MP has received consulting fees/honoraria and has served as a speaker or advisory board member for Amgen, Merck Serono, Roche, Lilly, MSD, BMS, Servier. DBV received speaker's honoraria from Roche, BMS, Pfizer, and Falk foundation, consultant's honoraria from Pfizer, Bristol Myers Squibb, and Gilead and travel support and congress registration fees from Gilead, Celgene, and Abbvie. All other authors declare that they have no competing interests.

## Author contributions

The study was designed by DBV and SAH. TMR, SLB, DBV, SAH performed the xenograft experiments. RT‐PCRs, western blots and gene expression analysis were done by TMR, SLB, AS, AM, and SAH. Tumour material was provided by MP, HW, RV, CT, AT, WS, and DBV. BV, STL, and AT performed Targeted Next Generation Sequencing. TL and SAH performed transcriptome analysis. Whole exome sequencing and data analysis was performed by JA and NC. The manuscript was drafted by TMR, SAH, and DBV. All authors read and approved the final manuscript.

### Peer review

The peer review history for this article is available at https://www.webofscience.com/api/gateway/wos/peer‐review/10.1002/1878‐0261.13510.

## Supporting information


**Table S1.** Mutations detected by targeted sequencing used for model selection.
**Table S2.** Mutations detected by exome sequencing and filtered by the Cancer.
**Table S4.** CMS classification of untreated control tumours.
**Fig. S1.** Overview of targeted sequencing results.
**Fig. S2.** Response data for different anti‐EGFR antibodies and MEK inhibitors tested in BoC105 and BoC147.
**Fig. S3.** Growth curves and waterfall plots of additional PDX models.
**Fig. S4.** Growth curves and waterfall plots of PDX models showing partial response (combination therapy only).
**Fig. S5.** Growth curves and waterfall plots of PDX models showing stable disease (combination therapy only).
**Fig. S6.** Growth curves and waterfall plots of PDX models showing progressive disease (combination therapy only).
**Fig. S7.** Additional tumours not shown in Fig. 3 receiving intermittent treatment.
**Fig. S8.** Western Blots of the first set of PDX models tested.
**Fig. S9.** Assessment of ERK and AKT phosphorylation in resistant and responding tumours.
**Fig. S10.** Whole exome sequencing results.

**Table S3.** Summary of gene expression data for each individual xenograft tumour analysed filtered for KEGG MAPK pathway genes (p 0.05).

## Data Availability

Growth curves are widely available in the supplementary material. Primary data will be made available on request by the corresponding authors. Whole exome sequencing has been deposited into the NCBI BioProject database under the BioProject ID PRJNA 988418 (Reviewer Link: https://dataview.ncbi.nlm.nih.gov/object/PRJNA988418?reviewer=ehlehc7iirecmffhm6cgisj2c8).Transcriptome data are available at GEO database (accession number GSE236078; https://www.ncbi.nlm.nih.gov/geo/query/acc.cgi?acc=GSE236078).
