# Supplementary material for: Lasting response by vertical inhibition with cetuximab and trametinib in KRAS‐mutated colorectal cancer patient‐derived xenografts
Source: Mol Oncol. 2023 Sep 3;17(11):2396–414. doi: 10.1002/1878-0261.13510 (PMC10620118; doi:10.1002/1878-0261.13510)
Supplement: Supplementary file 1 — Table S1. Mutations detected by targeted sequencing used for model selection. Table S2. Mutations detected by exome sequencing and filtered by the Cancer. Table S4. CMS classification of untreated control tumours. Fig. S1. Overview of targeted sequencing results. Fig. S2. Response data for different anti‐EGFR antibodies and MEK inhibitors tested in BoC105 and BoC147. Fig. S3. Growth curves and waterfall plots of additional PDX models. Fig. S4. Growth curves and waterfall plots of PDX models showing partial response (combination therapy only). Fig. S5. Growth curves and waterfall plots of PDX models showing stable disease (combination therapy only). Fig. S6. Growth curves and waterfall plots of PDX models showing progressive disease (combination therapy only). Fig. S7. Additional tumours not shown in Fig. 3 receiving intermittent treatment. Fig. S8. Western Blots of the first set of PDX models tested. Fig. S9. Assessment of ERK and AKT phosphorylation in resistant and responding tumours. Fig. S10. Whole exome sequencing results. [file MOL2-17-2396-s001.pdf]

Lasting response by vertical inhibition with cetuximab and trametinib in *KRAS*-mutated colorectal cancer patient-derived xenografts

Timm M. Reissig[1,2,3], Swetlana Ladigan-Badura[1,4], Anja Steinberg[1], Abdelouahid Maghnouj[1], Ting Li[1], Berlinda Verdoodt[5], Sven T. Liffers[3,5], Michael Pohl[4], Heiner Wolters[6], Christian Teschendorf[6], Richard Viebahn[7], Jakob Admard[8], Nicolas Casadei[8], Andrea Tannapfel[5], Wolff Schmiegel[4], Stephan A. Hahn[1*], Deepak B. Vangala[1,4*]

Supplementary Figures and Tables

Legends:

Suppl. Table 1: Mutations detected by targeted sequencing used for model selection. For an overview regarding wild-type status also see Suppl. Figure 1.

Suppl. Table 2: Mutations detected by exome sequencing and filtered by the Cancer Mutation Census tier 1 to 3 criteria. See also Suppl. Figure 1 for pre-treatment mutation status determined via targeted sequencing for model selection.

Suppl. Table 3: Summary of gene expression data for each individual xenograft tumor Analyzed and filtered for KEGG MAPK pathway genes (p 0.05). 5dCT – 5 days of treatment with cetuximab and trametinib, CTR – cetuximab and trametinib resistant tumor. (see separate Excel File).

Suppl. Table 4: CMS classification of untreated control tumors.

Suppl. Figure 1: Overview of targeted sequencing results
For mutational analysis, a gene panel with 48 genes (212 Amplicons) was utilized. The mutational status of the 19 models included in the study is depicted. For details regarding the variants found see Suppl. Table 1.

Suppl. Figure 2: Response data for different anti-EGFR antibodies and MEK inhibitors tested in BoC105 and BoC147.
Indicated numbers of BoC105 (A-C) and BoC147 (D-F) PDX tumors were treated with either mono or combination treatments. *, $p < 0.05$, **, $p < 0.01$, ***, $p < 0.001$. PR, partial response; PD, progressive disease. Each # indicates the last point in time a tumor was measured and taken out therafter.

Suppl. Fig. 3: Growth curves and waterfall plots of additional PDX models
Relative growth curves are derived from mean values ± SEM (error bars). Each # represents a tumor that was taken out of the treatment cohort at the indicated time point either because the tumor reached the maximum size criteria or due to health issues of the animal. Vertical gray bars indicate the end of the primary observational period of 28 days. Waterfall plots show the response after 59 days of treatment or before the end of the experiment compared with tumor volume at baseline. Each bar represents one tumor. Dotted lines indicate the cut-

off values for progressive disease (PD) and partial response (PR). *, $p < 0.05$, **, $p < 0.01$, ***, $p < 0.001$.

Suppl. Fig. 4: Growth curves and waterfall plots of PDX models showing partial response (combination therapy only)
Each # indicates the last point in time one tumor was measured and taken out thereafter. Vertical gray bars indicate the end of the primary observational period of 28 days.Waterfall plots show the response after 59 days of treatment or before the end of the experiment compared with tumor volume at baseline. Each bar represents one tumor. Dotted lines indicate the cut-off values for progressive disease (PD) and partial response (PR). *, $p < 0.05$, **, $p < 0.01$, ***, $p < 0.001$.

Suppl. Fig. 5: Growth curves and waterfall plots of PDX models showing stable disease (combination therapy only)
Each # indicates the last point in time one tumor was measured and taken out thereafter. Vertical gray bars indicate the end of the primary observational period of 28 days.Waterfall plots show the response after 59 days of treatment or before the end of the experiment compared with tumor volume at baseline. Each bar represents one tumor. Dotted lines indicate the cut-off values for progressive disease (PD) and partial response (PR). *, $p < 0.05$, **, $p < 0.01$, ***, $p < 0.001$.

Suppl. Fig. 6: Growth curves and waterfall plots of PDX models showing progressive disease (combination therapy only)
Each # indicates the last point in time one tumor was measured and taken out afterwards. Vertical gray bars indicate end of the primary observational period of 28 days. Waterfall plots show the response after 59 days of treatment or before end of experiment compared with tumor volume at baseline. Each bar represents one tumor. Dotted lines indicate the cut-off values for progressive disease (PD) and partial response (PR). *, $p < 0.05$, **, $p < 0.01$, ***, $p < 0.001$.

Suppl. Figure 7: Additional tumors not shown in Fig. 3 receiving intermittent treatment
In tumors responding until day 59, treatment was paused until tumor volume reached at least 200mm^3 again. In case of disease control upon retreatment for at least 30 days, the treatment was paused again. Treatment was re-initialized if the tumor regrew (200mm^3 in case of partial response, 400mm^3 in case of stable disease). The treatment pauses are highlighted in grey, treatment periods in blue. Only one tumor of the BoC137 PDX model showed secondary resistance already in the second treatment cycle (A, right panel, top growth curve).

Suppl. Fig. 8: Western Blots of the first set of PDX models tested

C, untreated control tumor; 5dCT, tumor harvested after combination therapy for 5 days.

Suppl. Fig. 9: Assessment of ERK and AKT phosphorylation in resistant and responding tumors

Phosphorylation of ERK and AKT in primarily resistant tumors (A), secondarily resistant tumors (B) and tumors with partial response (C). C, control tumor; 5dCT, tumor harvested after combination therapy for 5 days; CT, tumor harvested at the end of combination therapy. Most resistant tumors show an increase in ERK-phosphorylation at the end of treatment, suggesting pathway re-activation.

Suppl. Fig. 10: Whole exome sequencing results

BoC51, BoC109, BoC117, and BoC122 were primary resistant to vertical inhibition with cetuximab and trametinib (suffix CT). Secondary resistant tumors BoC2 and BoC56 were compared to control tumors (suffix K).

Suppl. Table 1

PDX	gene	variant	amino.acid
**BoC2**	APC	stop_gained	p.Q1367Ter
**BoC2**	HNF1A	frameshift_variant	p.P291QfsTer51
**BoC2**	KRAS	missense_variant	p.G12D
**BoC2**	STK11	frameshift_variant	p.P281RfsTer6
**BoC9**	APC	stop_gained	p.Q1378Ter
**BoC9**	HNF1A	frameshift_variant	p.P291QfsTer51
**BoC9**	KRAS	missense_variant	p.G12D
**BoC9**	TP53	missense_variant	p.R175H
**BoC14**	APC	stop_gained	p.R876Ter
**BoC14**	FBXW7	missense_variant	p.R399Q
**BoC14**	KRAS	missense_variant	p.A146T
**BoC14**	MET	missense_variant	p.T1010I
**BoC14**	PTEN	stop_gained	p.Q17Ter
**BoC19**	CTNNB1	missense_variant	p.S45F
**BoC19**	KRAS	missense_variant	p.A146T
**BoC19**	TP53	missense_variant	p.R175H
**BoC46**	FBXW7	missense_variant	p.R399Q
**BoC46**	KRAS	missense_variant	p.G13D
**BoC46**	TP53	missense_variant	p.R175H
**BoC47**	KRAS	missense_variant	p.G12D
**BoC47**	TP53	missense_variant	p.C141Y
**BoC51**	KRAS	missense_variant	p.G12D
**BoC56**	APC	stop_gained	p.Q1429Ter
**BoC56**	HNF1A	frameshift_variant	p.P291QfsTer51
**BoC56**	KRAS	missense_variant	p.G12C
**BoC64**	KRAS	missense_variant	p.G12D
**BoC64**	VHL	missense_variant	p.S111G
**BoC78**	KRAS	missense_variant	p.G12D
**BoC78**	TP53	missense_variant	p.P72R
**BoC80**	KRAS	missense_variant	p.G12V
**BoC105**	KRAS	missense_variant	p.G12V
**BoC105**	PIK3CA	missense_variant	p.E545K

**BoC109**	KRAS	missense_variant	p.G13D
**BoC117**	KRAS	missense_variant	p.G12D
**BoC122**	APC	stop_gained	p.E1379Ter
**BoC122**	FBXW7	missense_variant	p.R505C
**BoC122**	KRAS	missense_variant	p.G12D
**BoC130**	APC	stop_gained	p.Q1367Ter
**BoC130**	KRAS	missense_variant	p.G12D
**BoC130**	TP53	missense_variant	p.R273H
**BoC136**	KRAS	missense_variant	p.G12V
**BoC137**	KRAS	missense_variant	p.G12D
**BoC147**	APC	stop_gained	p.R1114Ter
**BoC147**	KRAS	missense_variant	p.G12A
**BoC147**	TP53	missense_variant	p.R248Q

Suppl. Table 2

pdx_new2	response	gene	variant_type	protein_letter
**BoC122_CT5**	primary resistant	FBXW7	missense	p.R505C
**BoC122_CT5**	primary resistant	APC	stop_gained	p.E1379Ter
**BoC122_CT5**	primary resistant	KRAS	missense	p.G12D
**BoC117_CT7**	primary resistant	KRAS	missense	p.G12D
**BoC109_CT2**	primary resistant	CDC73	stop_gained	p.R9Ter
**BoC109_CT2**	primary resistant	KRAS	missense	p.G13D
**BoC109_CT2**	primary resistant	NF1	stop_gained	p.R826Ter
**BoC51_CT5**	primary resistant	KRAS	missense	p.G12D
**BoC2_K4**	untreated contr.	APC	stop_gained	p.Q1367Ter
**BoC2_K4**	untreated contr.	KRAS	missense	p.G12D
**BoC2_CT6**	secondary resistant	APC	stop_gained	p.Q1367Ter
**BoC2_CT6**	secondary resistant	KRAS	missense	p.G12D
**BoC56_K1**	untreated contr.	ACVR1	missense	p.G328V
**BoC56_K1**	untreated contr.	APC	stop_gained	p.Q1429Ter
**BoC56_K1**	untreated contr.	KRAS	missense	p.G12C
**BoC56 CT4**	secondary resistant	ACVR1	missense	p.G328V
**BoC56 CT4**	secondary resistant	APC	stop_gained	p.Q1429Ter
**BoC56 CT4**	secondary resistant	KRAS	missense	p.G12C

Supplement Table 4

Xenograft Model	overall response	prediction	d.CMS1	d.CMS2	d.CMS3	d.CMS4	p.value	FDR
BoC109_K5	primary resistant	CMS1	0.631601353	0.788431338	0.784025432	0.669195662	0.001	0.0018125
BoC137_K5	partial response	CMS1	0.669194477	0.681091723	0.678559053	0.710930112	0.007992008	0.012528013
BoC2_K3	secondary resistant	CMS1	0.640473134	0.713492363	0.699673808	0.723930893	0.001	0.0018125
BoC64_K1	primary resistant	CMS1	0.632896202	0.728554032	0.657721579	0.782731538	0.001	0.0018125
BoC117_K7	primary resistant	CMS2	0.777720818	0.648861808	0.711894881	0.808805662	0.001	0.0018125
BoC47_K7	partial response	CMS2	0.797619402	0.656023251	0.744769548	0.826778781	0.008991009	0.013723119
BoC51_K1	primary resistant	CMS3	0.628891996	0.79452842	0.593918342	0.669839805	0.001	0.0018125
BoC56_K1	secondary resistant	CMS3	0.727319498	0.6687746	0.616951422	0.632473103	0.001	0.0018125
BoC122_K3	primary resistant	CMS4	0.668496017	0.725570096	0.691137298	0.626319497	0.001	0.0018125

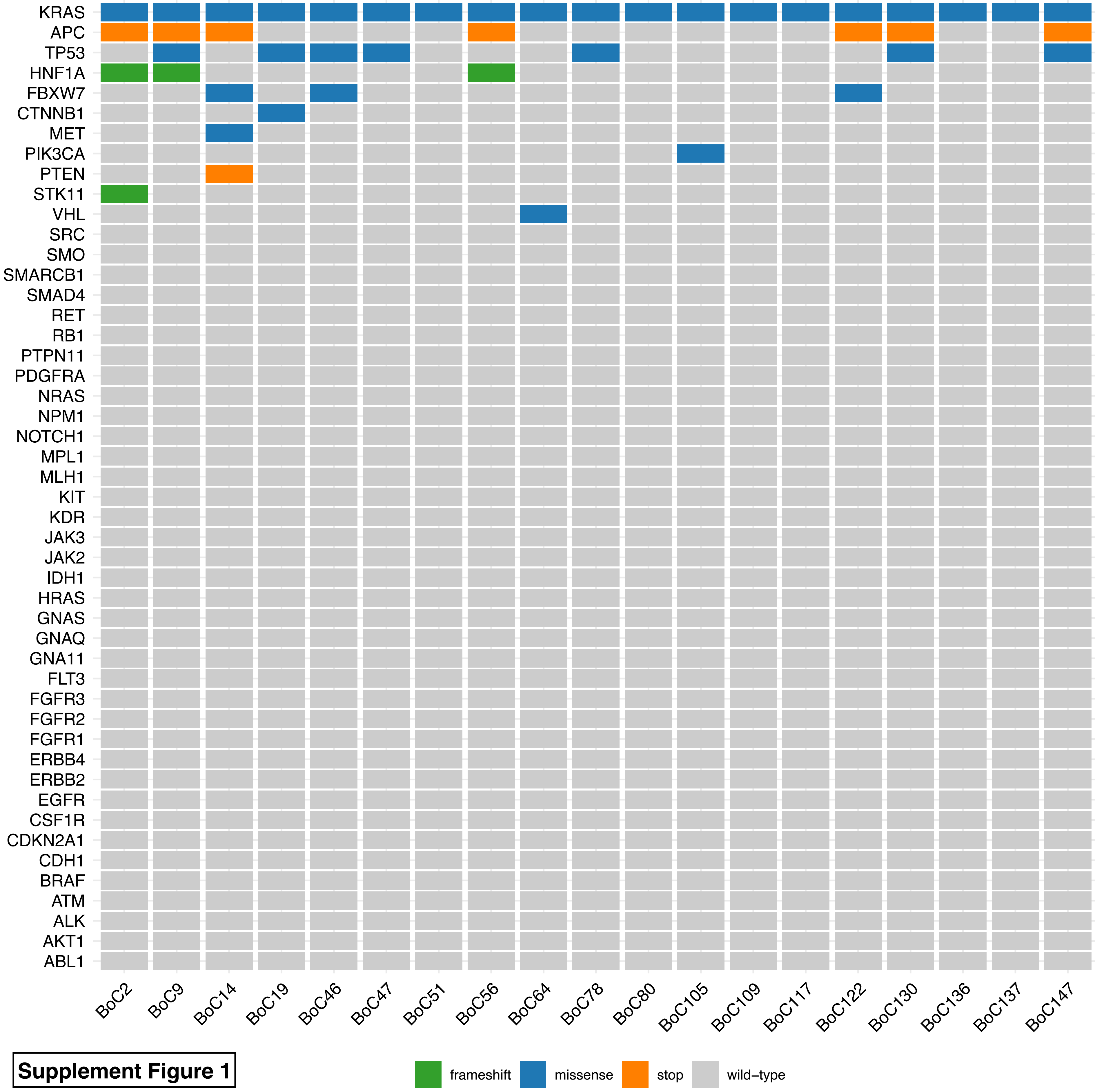

Supplement Figure 1

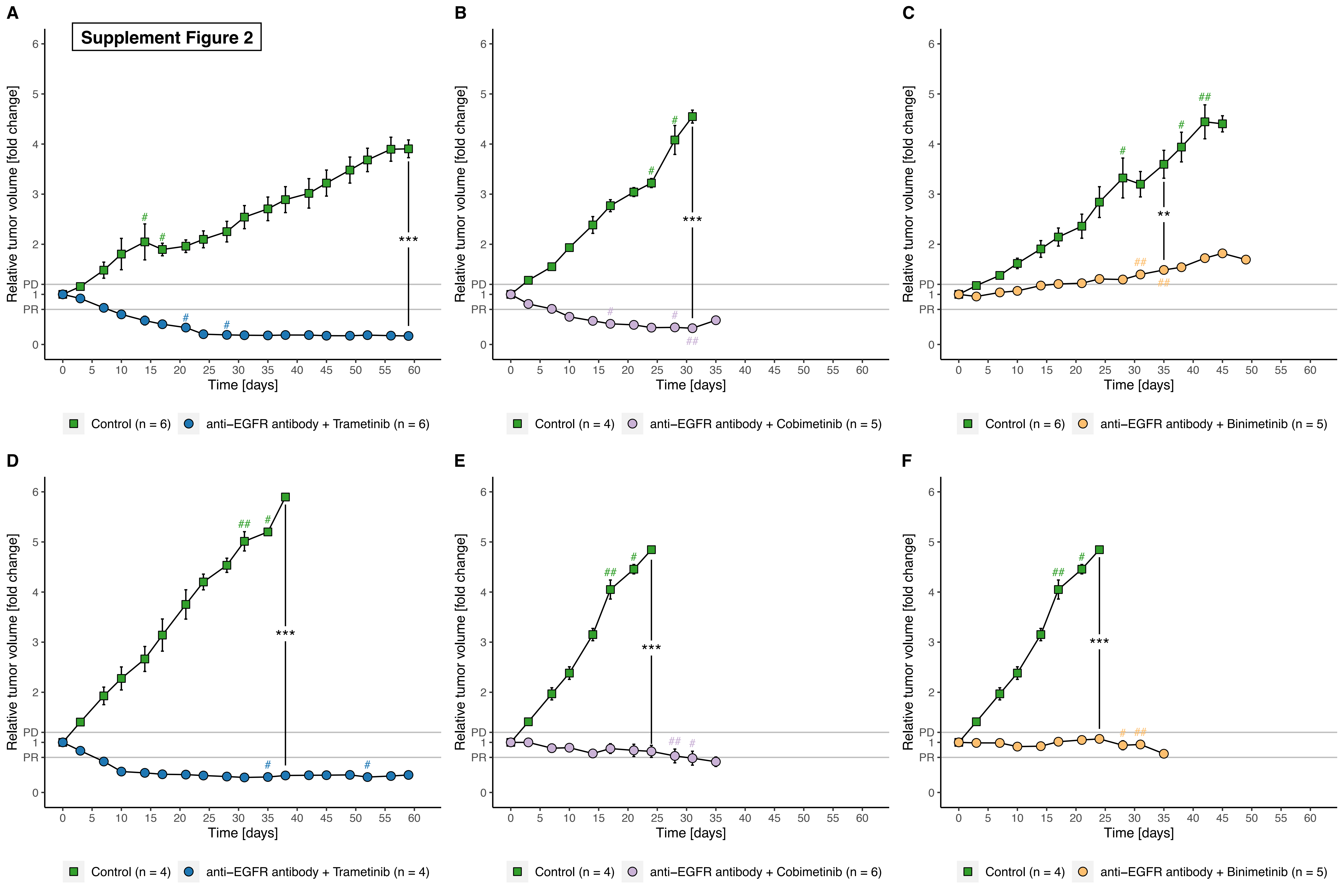

Supplement Figure 2
A
B
C
D
E
F
Relative tumor volume [fold change]
Time [days]
PD
PR
***
**
#
##
Control (n = 6)
anti−EGFR antibody + Trametinib (n = 6)
Control (n = 4)
anti−EGFR antibody + Cobimetinib (n = 5)
Control (n = 6)
anti−EGFR antibody + Binimetinib (n = 5)
Control (n = 4)
anti−EGFR antibody + Trametinib (n = 4)
Control (n = 4)
anti−EGFR antibody + Cobimetinib (n = 6)
Control (n = 4)
anti−EGFR antibody + Binimetinib (n = 5)

Supplement Figure 3

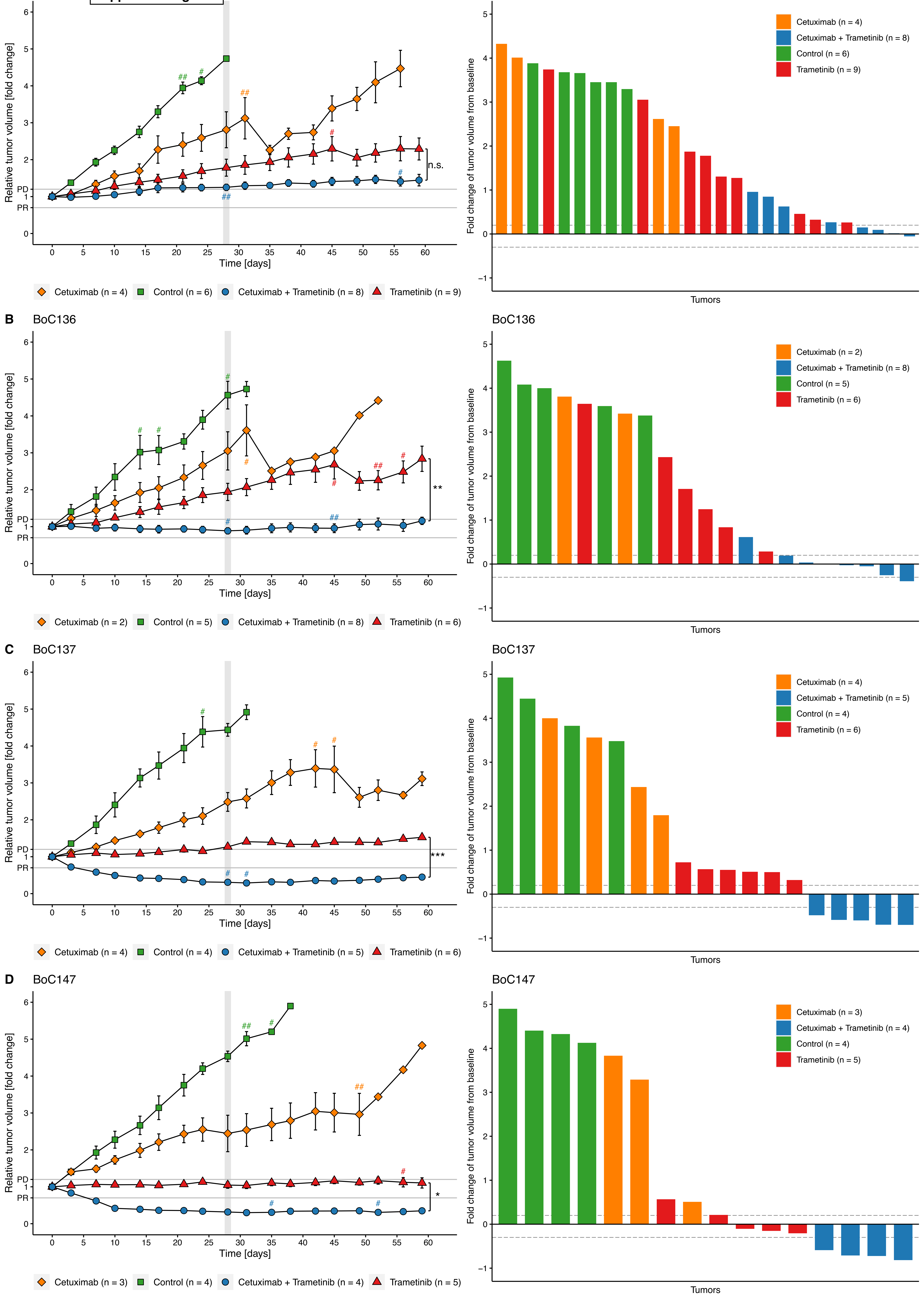

Supplement Figure 4

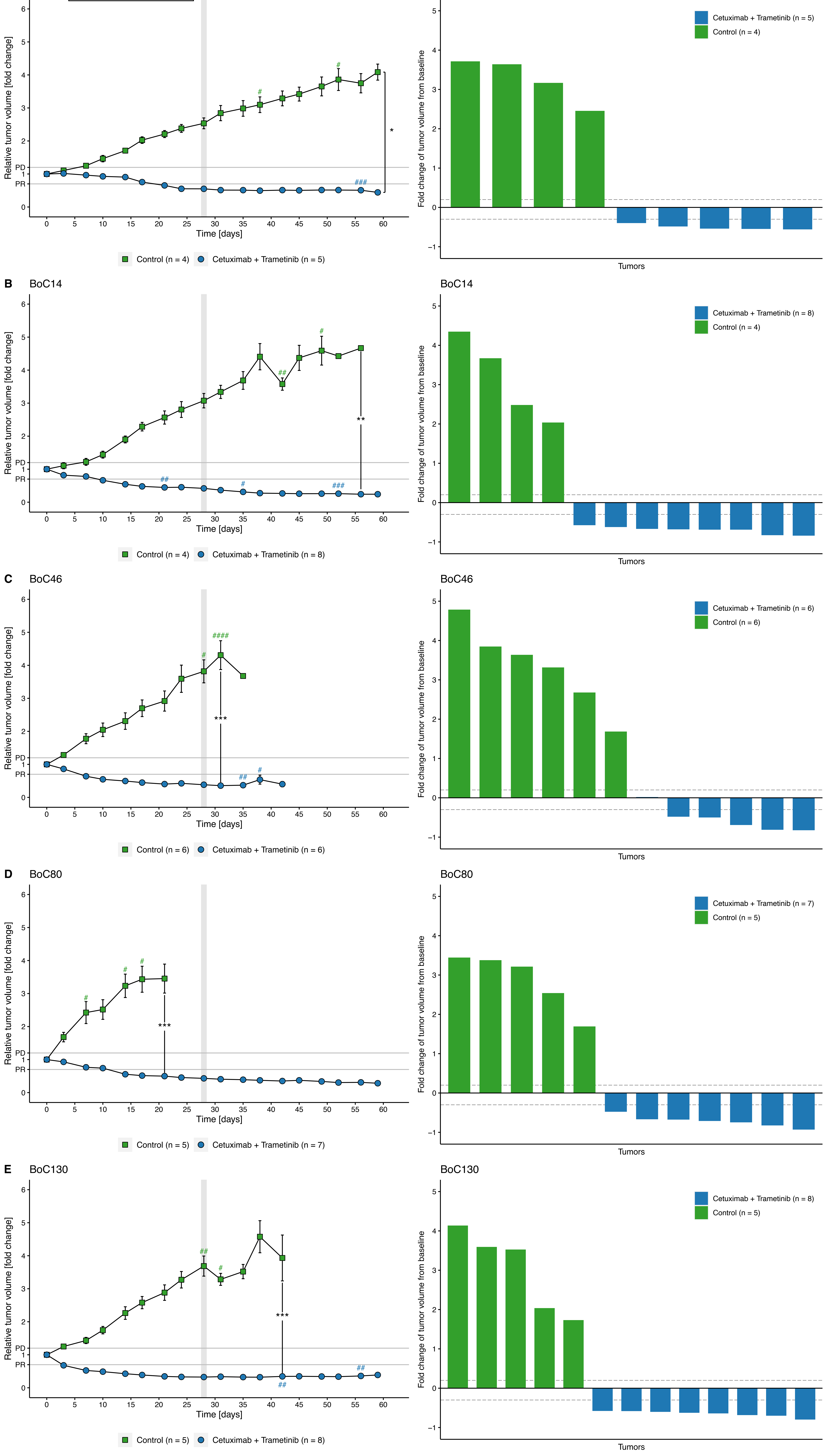

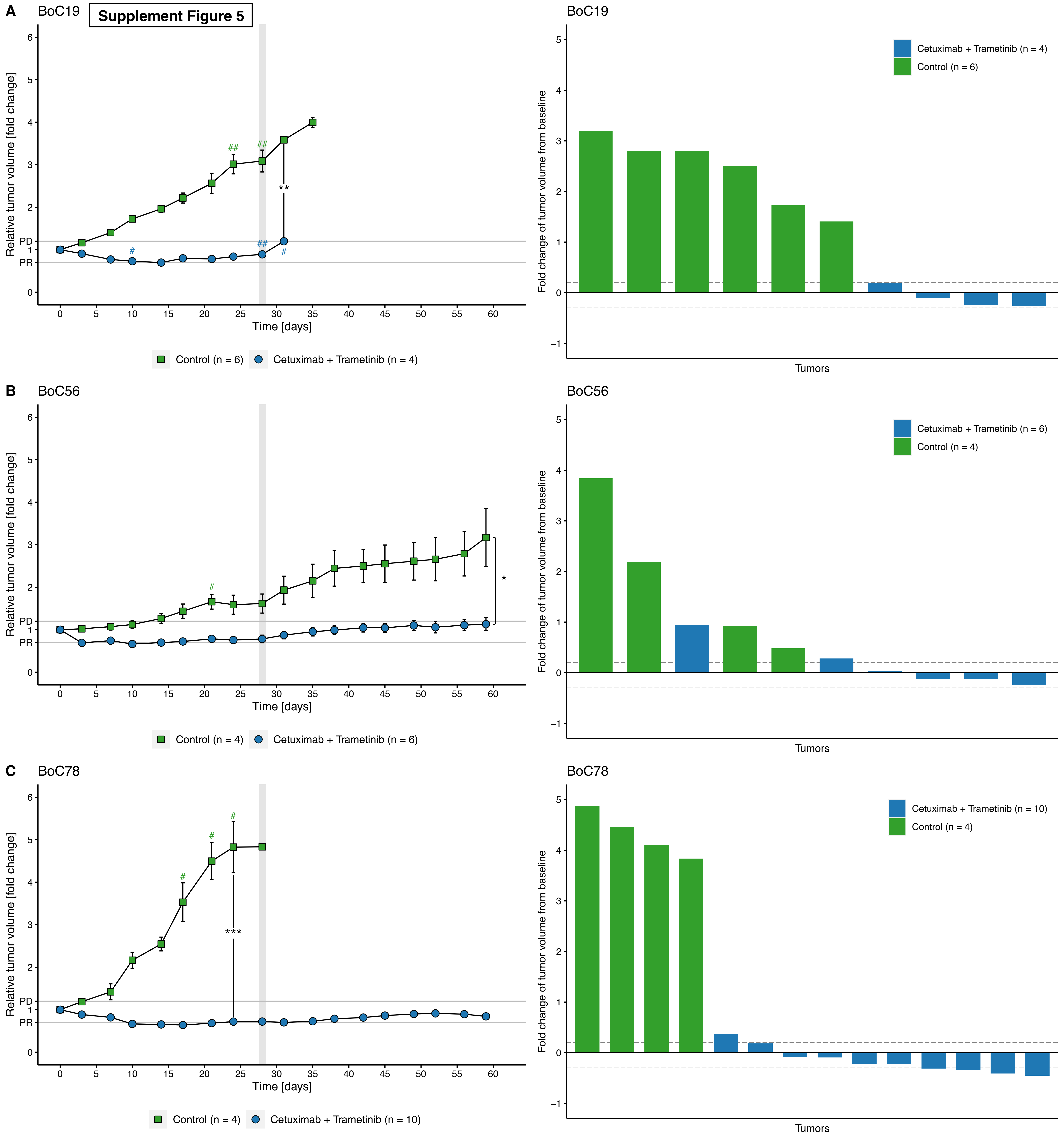
Supplement Figure 5
A
BoC19
Relative tumor volume [fold change]
Time [days]
PD
PR
##
#
**
Control (n = 6)
Cetuximab + Trametinib (n = 4)
BoC19
Fold change of tumor volume from baseline
Tumors
Cetuximab + Trametinib (n = 4)
Control (n = 6)
B
BoC56
Relative tumor volume [fold change]
Time [days]
*
Control (n = 4)
Cetuximab + Trametinib (n = 6)
BoC56
Fold change of tumor volume from baseline
Tumors
Cetuximab + Trametinib (n = 6)
Control (n = 4)
C
BoC78
Relative tumor volume [fold change]
Time [days]
***
Control (n = 4)
Cetuximab + Trametinib (n = 10)
BoC78
Fold change of tumor volume from baseline
Tumors
Cetuximab + Trametinib (n = 10)
Control (n = 4)

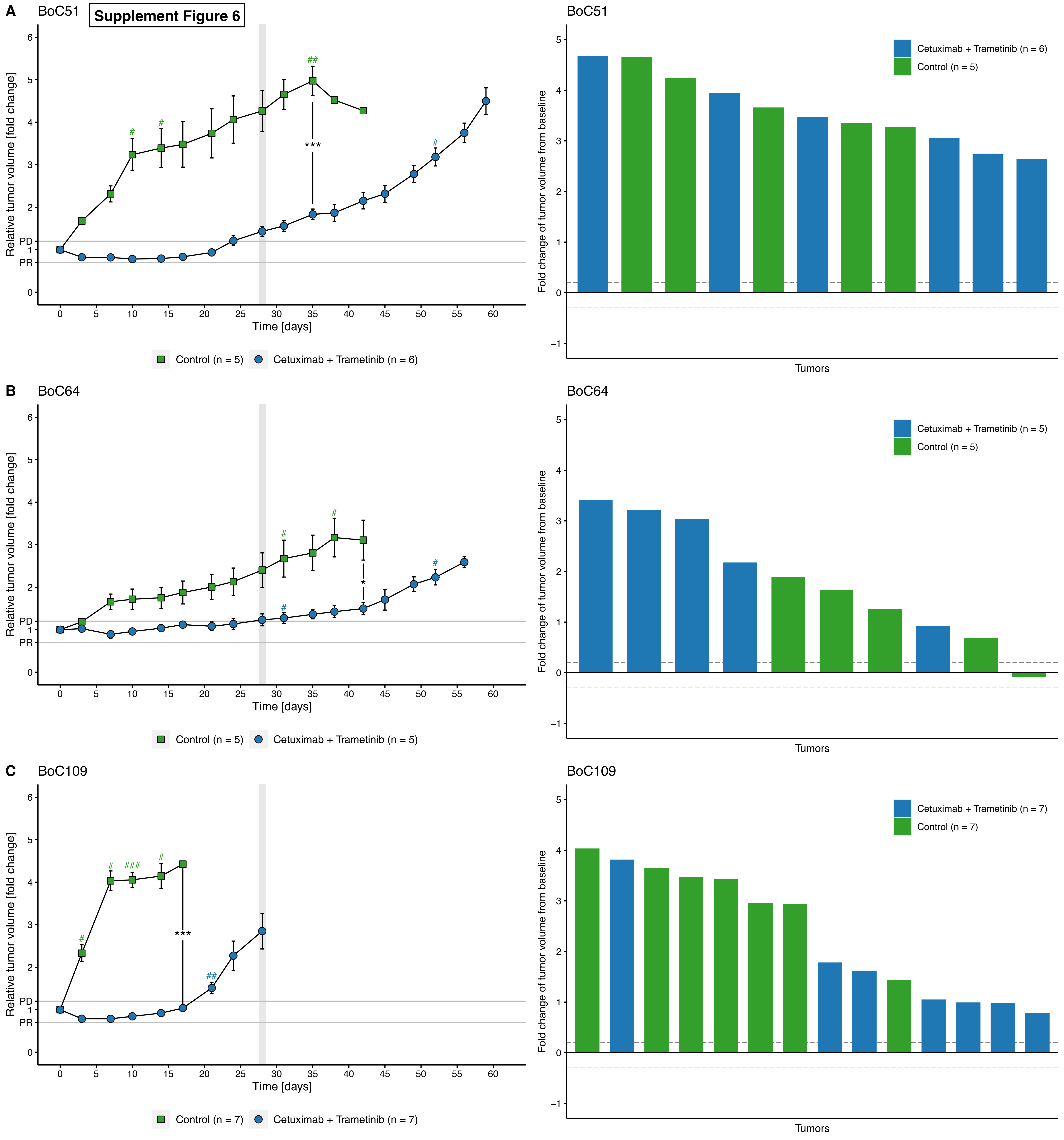
Supplement Figure 6
A
BoC51
Relative tumor volume [fold change]
Time [days]
Control (n = 5)
Cetuximab + Trametinib (n = 6)
PD
PR
***
BoC51
Fold change of tumor volume from baseline
Tumors
Cetuximab + Trametinib (n = 6)
Control (n = 5)
B
BoC64
Relative tumor volume [fold change]
Time [days]
Control (n = 5)
Cetuximab + Trametinib (n = 5)
BoC64
Fold change of tumor volume from baseline
Tumors
Cetuximab + Trametinib (n = 5)
Control (n = 5)
C
BoC109
Relative tumor volume [fold change]
Time [days]
Control (n = 7)
Cetuximab + Trametinib (n = 7)
BoC109
Fold change of tumor volume from baseline
Tumors
Cetuximab + Trametinib (n = 7)
Control (n = 7)

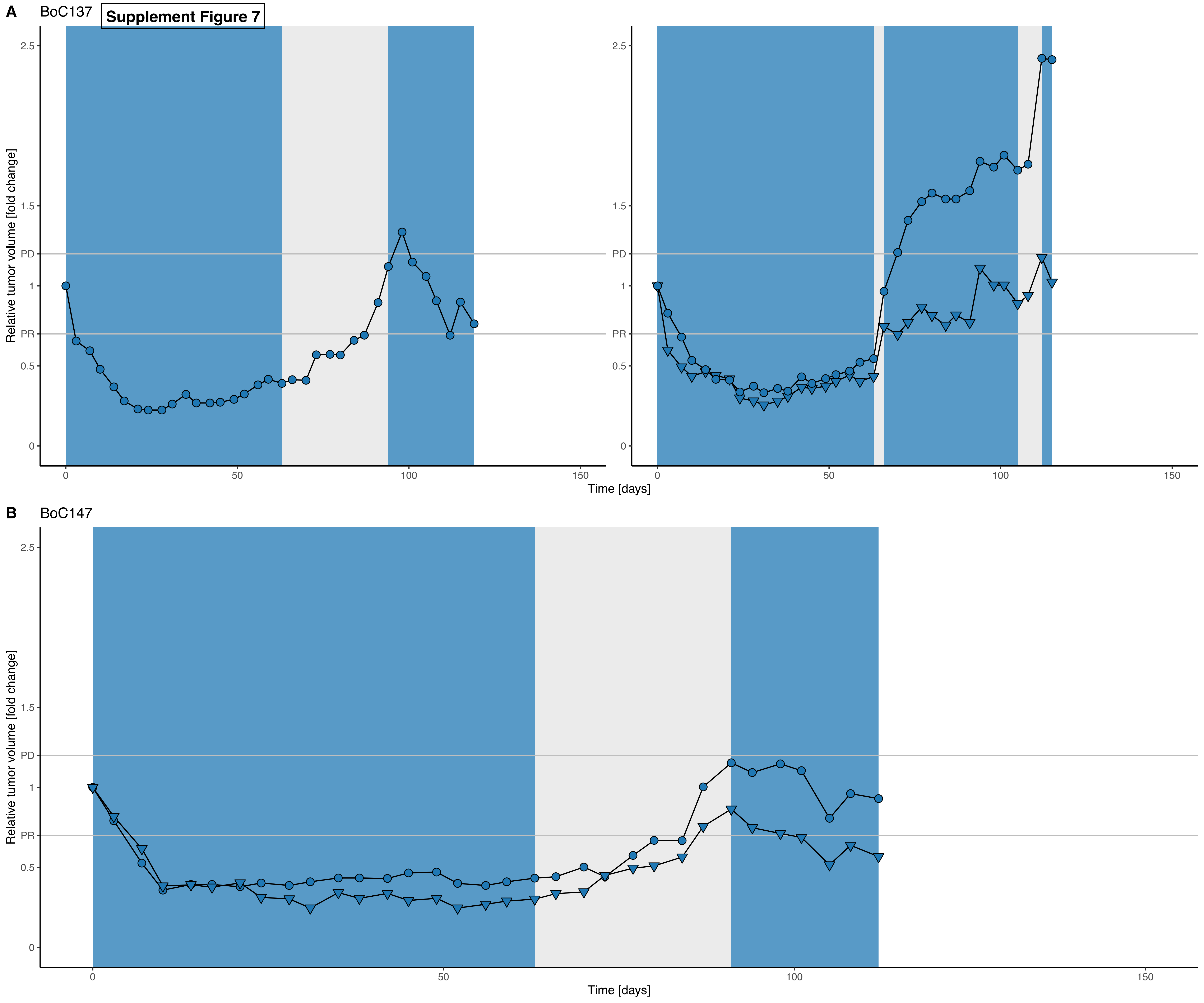

A
BoC137
Supplement Figure 7
B
BoC147
Relative tumor volume [fold change]
2.5
1.5
PD
1
PR
0.5
0
0
50
100
150
Time [days]

Supplement Figure 8

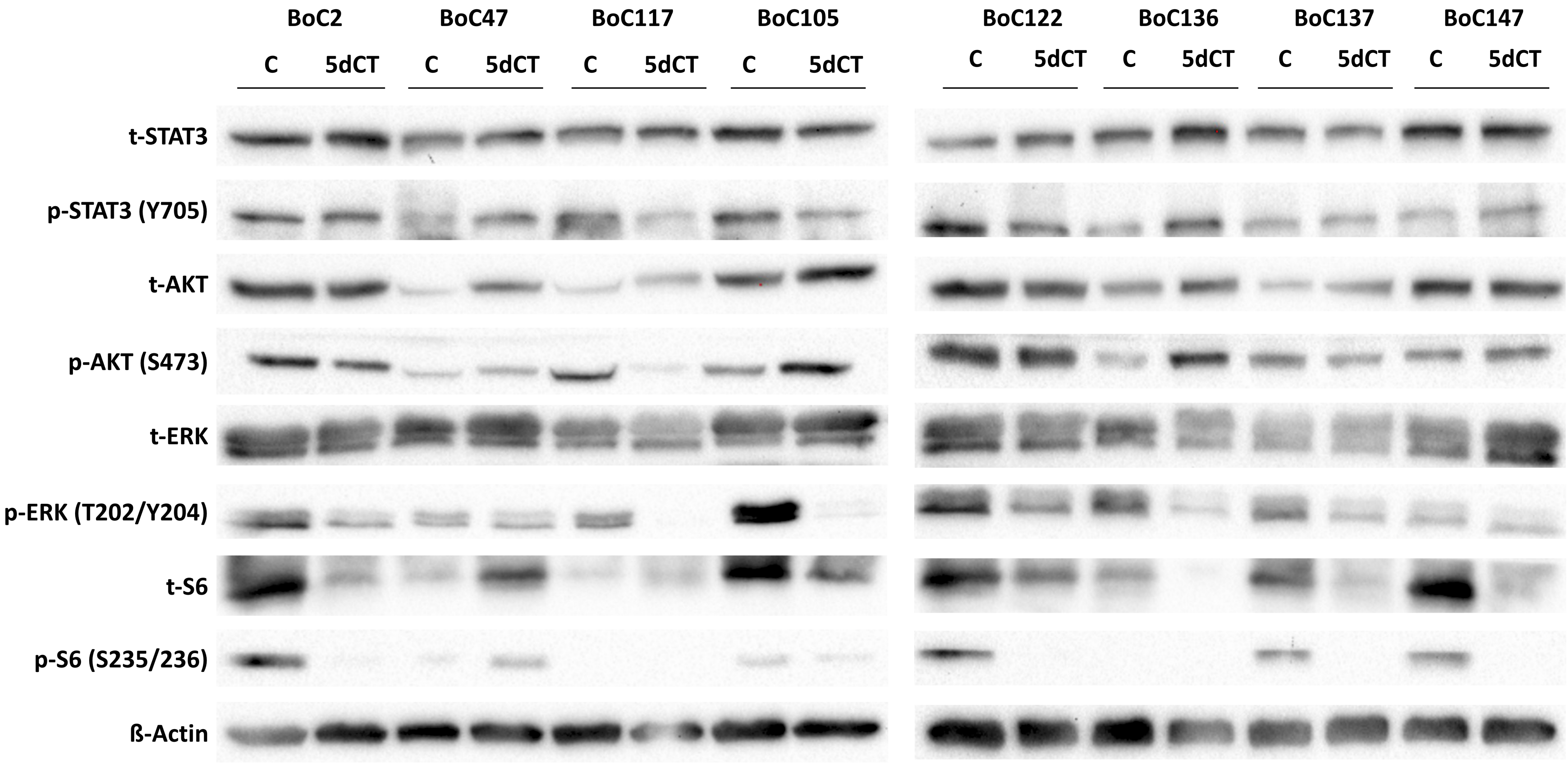

Supplement Figure 9

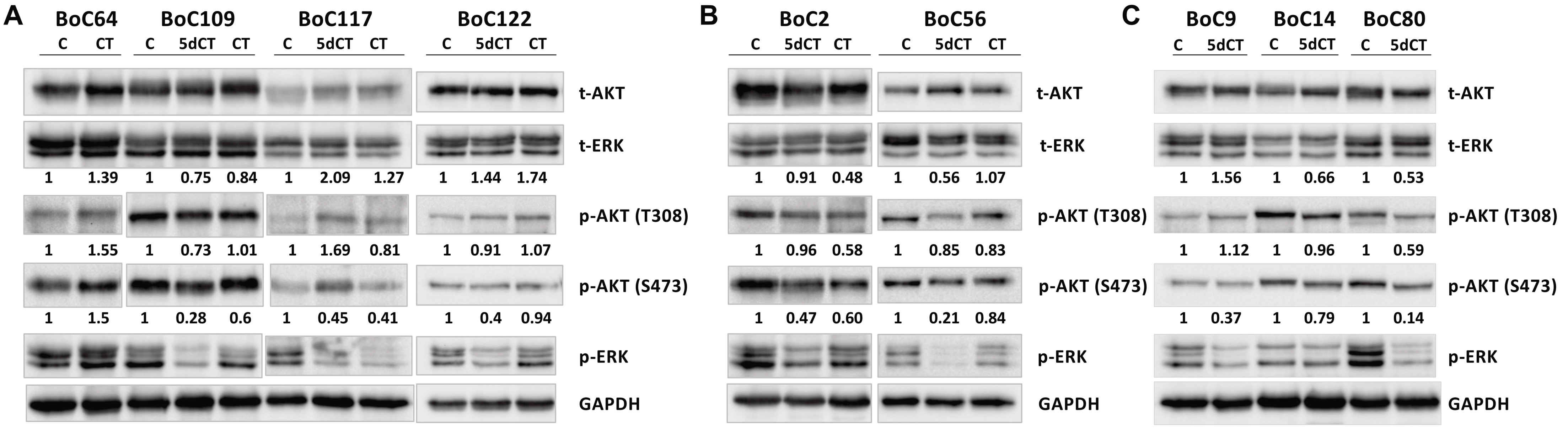

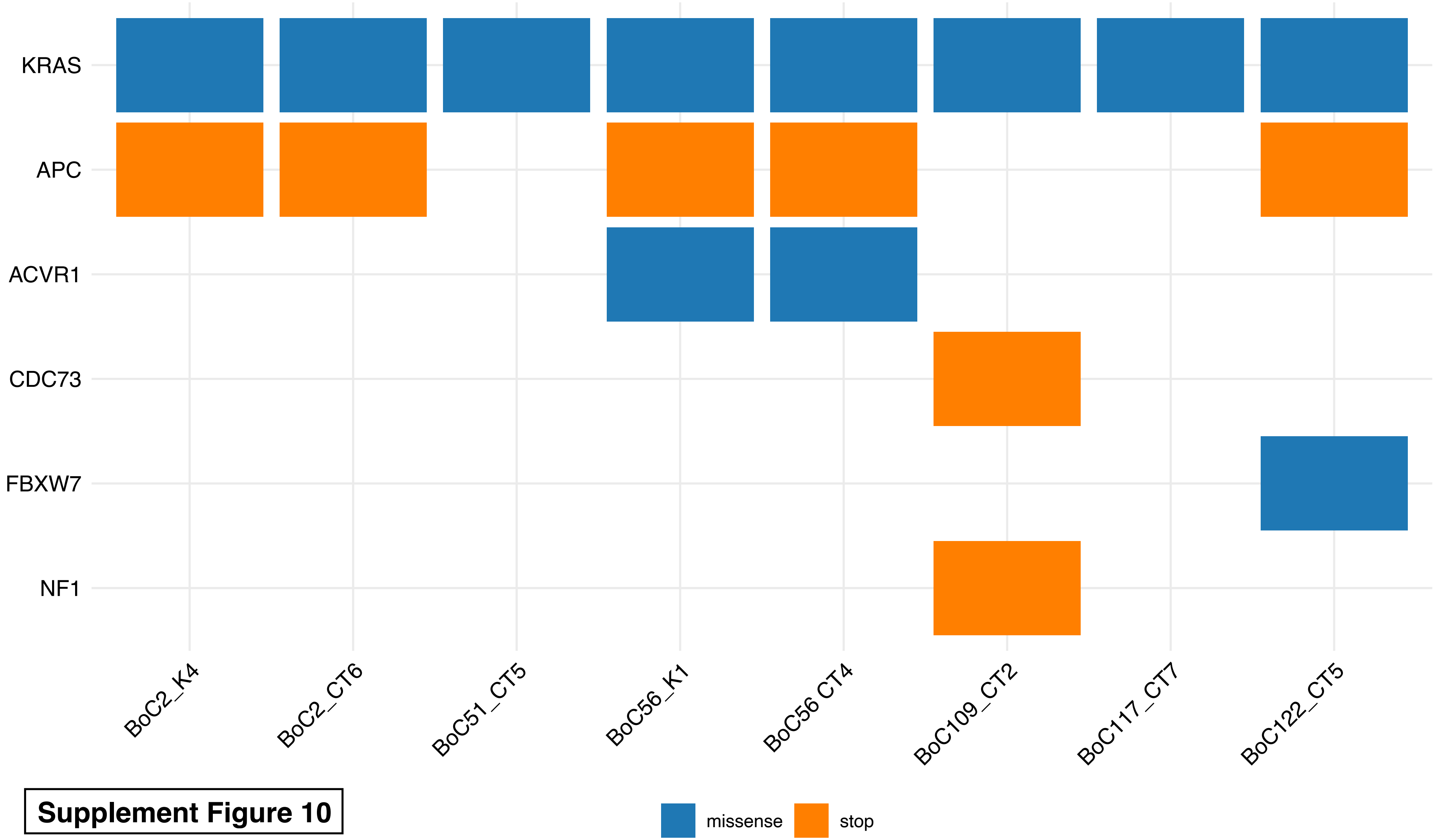

Supplement Figure 10
